# Association of compassion and empathy with prosocial health behaviors and attitudes in a pandemic

**DOI:** 10.1371/journal.pone.0271829

**Published:** 2022-07-22

**Authors:** Melissa M. Karnaze, John Bellettiere, Cinnamon S. Bloss

**Affiliations:** 1 Herbert Wertheim School of Public Health and Longevity Science, UC San Diego, La Jolla, CA, United States of America; 2 Center for Empathy and Technology, T. Denny Sanford Institute for Empathy and Compassion, University of California, San Diego, La Jolla, CA, United States of America; Universitat de Valencia, SPAIN

## Abstract

This investigation examined how dispositional compassion and empathy were associated with prosocial behaviors and attitudes in the SARS-CoV-2 pandemic. Every two weeks from March 22 to June 15, 2020, we fielded a survey to a new cohort of adults in the U.S. Compassion related to whether one stayed home to protect others, more hours spent staying home and distancing from others, and more frequent mask wearing in public, in the past two weeks. Compassion also related to greater perceived ability to help others who were negatively affected. Empathy related to more endorsement of understanding others’ fear of COVID-19, and less endorsement of the view that others were overreacting to COVID-19. There was an interaction between empathy and political ideology, suggesting that empathy may matter for understanding others’ fear among those with more conservative-leaning beliefs. Empathy also related to greater understanding that sheltering-in-place helps prevent the spread of COVID-19. Findings suggest that messaging and interventions to increase compassion and empathy may promote public health behaviors during a pandemic regardless of political orientation. Targeting empathy may be one way to reach individuals with more conservative political beliefs, and it is important to use an evidence-based approach accounting for political party differences in motivated reasoning.

## Introduction

After collective adverse events, people often come together to help victims survive the aftermath. People can also perceive positive social benefits after tragedy, such as increased prosocial behavior and connectedness [1]. Catastrophes may facilitate prosocial responses by unifying people and helping them focus on a common cause [2]. However, it is less clear whether a pandemic would motivate people around a common cause as the opposition, or virus, is often undetectable. Thus, people can fear others, as they may carry the invisible virus, and uncertainty concerning the pandemic’s duration or societal impacts can contribute to distress and social unrest [3, 4]. Yet, people do show prosocial responses during pandemics [5]. We propose that two prosocial tendencies in normal day-to-day life, having compassion and empathy for others, should predict prosocial responses during a pandemic [2].

### Compassion and empathy

Compassion is commonly defined as an affective state that includes the motivation to help those who are suffering or in need, and it has been associated with helping behaviors even if those behaviors incur personal cost [6]. Empathy is related to compassion but it is a broader construct [7]. Empathy is commonly defined as the ability to understand and experience the internal states of others [8]. In addition to experiencing empathy for others when they are visibly distressed, people can also do so when others experience other emotional states, including positive ones. This ability is thought to be conducive to social relationships, as empathy is associated with more supportive and satisfying relationships with others [9–11]. Individual differences in compassion and empathy in daily life should matter for whether people engage in preventive health behaviors, particularly during a pandemic, when preventive behaviors not only protect one’s own health, but also protect others’ health by reducing viral transmission.

### Compassion, empathy, and responses to the pandemic

Some work has shown that compassion is related to more prosocial responses to the pandemic. One study found that people with more prosocial behavior prior to the pandemic reported more intentions to engage in preventive health behaviors early in the pandemic [12]. In a nationally representative sample, compassion for people with COVID-19 was associated with greater endorsement of government response measures to prevent transmission of the virus [13]. Another study looked at a latent construct referred to as “prosocial tendencies”, which predicted health behaviors such as mask wearing [14]. A study that focused on empathy found that empathic concern, or a subset of empathy referring to feelings of concern for others in distress, was correlated with the belief that wearing a mask in public is the right thing to do [15]. However, empathic concern was not associated with actual mask wearing behavior. Together, these findings suggest that compassion and empathy may enhance prosocial responses during a pandemic, but research is needed to determine whether they predict actual health behaviors.

Studies have shown that specific messaging can elicit empathy for those who are most vulnerable to the virus and increase intentions to engage in more physical distancing practices [16], and that empathy for those who are especially vulnerable to COVID-19 has been related to more self-reported social distancing [17] as well as more favorable attitudes about lockdown measures [18]. However, these studies lacked conceptual clarity on the distinctions between compassion and empathy as they assessed empathy using a brief measure containing items referring to both compassion and empathy (i.e., one of the three items referred to feeling “compassion” rather than empathy, and one item referred to feeling “moved”, which could be relevant to both compassion and empathy).

In the present investigation, we were deliberate in assessing both trait compassion and trait empathy, or tendencies to experience these prosocial states in daily life, using scales psychometrically validated prior to the pandemic. We also assessed reports of actual health behaviors rather than intentions. We expected that trait compassion would be related to behaviors, whereas trait empathy would be related to attitudes.

### Theories of compassion

According to evolutionary theories, compassionate responding to others in need would have been beneficial to ancestral humans for childrearing and social cohesion among groups [6]. In modern times, the ability to care for others in need should confer the same benefits, but people can also engage in prosocial behaviors that help others at potential personal cost. The tendency to have compassion for others in need and compassionate rather than self-image goals has been associated with indices of better social connections, such as perceived social support [19] and less loneliness [20].

Compassion should have implications in the context of responses to a collective stressor, such as a pandemic. Cognitive health behavior theories posit that people make decisions about whether to engage in preventive health behaviors by weighing the potential costs against potential benefits. In support of this theoretical framework, research prior to the SARS-CoV-2 pandemic showed that decisions to social distance during a pandemic involve just such a cost-benefit analysis [21]. Compassion involves prioritizing the needs of others who are suffering and thus feeling motivated to act in their interests, even at potential personal cost. Thus, we would expect that more compassionate individuals would be more likely to care about the health and safety of others during a pandemic and thus be more likely to behave in ways that prevent harm by minimizing chances of spreading the virus to them, even if at personal expense (e.g., staying home but missing out on social opportunities). Compassion should also shape the ways in which individuals construe the “costs” and “benefits” related to preventive health behaviors. From the perspective of more compassionate individuals, engaging in preventive health behaviors should be construed more as a benefit, given the value placed on promoting the health and wellbeing of others. Similarly, failure to engage in preventive health behaviors should be construed more as a cost, as it could cause personal distress to potentially worsen the health of others [22].

Social norms theory [23] also provides a rationale for why more compassionate individuals would engage more in preventive behaviors during a pandemic. Early in the pandemic, there were public calls for compassion and empathy. In the U.S., before the first shelter-in-place order, people were encouraged in many instances to stay home primarily to “save lives” and were later told to wear a mask to “protect others”. These appeals emphasized the social good resulting from engaging in such behaviors (even though they would also benefit in terms of their personal health), and thus emphasized injunctive norms, or perceptions of what is commonly approved of within one’s culture [24]. While interventions designed to influence health behaviors through injunctive norms are intended to have broad persuasion effects, more compassionate individuals, who are more attuned to the needs of others and collective wellbeing, should be more likely to change their behaviors in line with such injunctive norms. Thus we would expect that more compassionate individuals would be receptive to the early public health calls to engage in preventive behaviors for the benefit of fellow humans.

#### Hypotheses concerning dispositional compassion

We hypothesized that individual differences in compassion would be associated with greater engagement in actual behaviors that would benefit the health of others in the context of a pandemic, including social distancing and wearing a mask while in public.

### Theories of empathy

In addition to examining the role of compassion during a pandemic, it is also important to investigate whether individual differences in empathy are related to prosocial attitudes. Empathy is a key component of compassionate responding to others [22, 25]. Based on theories of compassion and empathy, without the first step of recognizing another’s distress (which empathy facilitates), the later step of feeling compassion, or motivation to relieve another’s distress, cannot occur. So, empathy may be an important domain for intervention as it may be able to enhance the ability to connect with others’ feelings over the long-term, even if they are negative, and in turn recognize and feel compassion for others’ who are suffering, and not just those who have similar experiences in the response to the pandemic (or similar political views about the pandemic).

While empathy may play a unique role in sustaining compassion during a pandemic in the long-term, it is important to note that in the shorter-term empathy should also be more strongly related to beliefs about others, rather than actions taken for the benefit of others (for which compassion should play a stronger role). In their model of translating empathy to prosocial behavior, Stevens and Taber [22] proposed that sharing the feelings of others (empathy) is necessary for caring about relieving others’ distress (empathic concern). However, self-regulation processes following empathy can either (a) promote empathic concern and thus compassion for others, or in contrast, (b) result in too much personal distress and lead to self-protective rather than prosocial behaviors [26]. Support for this model comes from experiments showing that compassion training decreases negative affect associated with seeing others in distress, and increases positive affect, which is a feature of compassion, as it involves positive regard for a person in distress and a desire to help them [27, 28]. Further support for this model comes from studies showing that while empathy may precede compassion, it is compassion rather than empathy that predicts whether individuals take prosocial actions to help others in distress [29, 30]. Thus, while empathy may be a necessary component of compassionate responding to others distress, compassion has been more strongly tied to action. In the context of a pandemic, when fear and other negative affective experiences can be widespread, the tendency to share the feelings of others (empathy) could result in empathic distress over time, which would not translate to more engagement in health behaviors. Thus, we did not expect that individual differences in empathy would be associated with early engagement in prosocial behaviors. Instead, we expected that early in the pandemic, more empathy for others in general would translate to more empathy in the context of the pandemic, in terms of understanding of others’ reactions to the pandemic.

In addition, we expected that more empathy for others in general would translate to better understanding of how one’s own engagement in a preventative health behavior would have implications for others’ health during the pandemic. According to the Health Belief Model [31], a cognitive health behavior theory, decisions about whether to engage in preventive health behaviors involve response efficacy beliefs, or beliefs that certain behaviors will minimize health risks. People who tend to have more empathy for others in daily life should be better able to see how their own health behaviors are connected to the health outcomes of others. Therefore, they should be better able to understand that a preventive health behavior, such as staying home during a pandemic, would reduce health risks for others. It is important to explore whether a relationship exists between empathy and this response efficacy belief, because over time, such beliefs may be key to sustaining voluntary health behaviors, like social distancing, that are protective for one’s own health as well as others’ health.

#### Hypotheses concerning dispositional empathy

We hypothesized that the tendency to have empathy for others in daily life would be associated with the extent to which individuals could understand and validate others’ affective responses concerning the pandemic, as well as greater understanding that a preventive health behavior during the pandemic (staying home) would have implications for the health of others (preventing the spread of COVID-19).

### The present investigation

Prior work exploring the role of compassion and empathy during the SARS-CoV-2 pandemic has been limited. First, many studies have relied on non-validated measures of empathy or compassion. Second, studies have focused more on intentions to engage in behaviors rather than actual behaviors. Third, studies have occurred later in the pandemic. To understand the role of dispositional compassion and empathy in the context of a pandemic, research is needed that uses validated measures of compassion and empathy to determine whether individual differences in these tendencies are associated with actual behaviors during a pandemic. It is also important to understand the role of compassion and empathy in the early phase of a pandemic, during which health behaviors can dramatically improve public health outcomes, and during which individual-level differences may have more of an impact than other factors, such as social group norms, including political partisanship. Most of the published literature in the context of the pandemic has focused on protective behavioral intentions and studies performed during such a critical period are rare.

In addition, any study of health behaviors during the SARS-CoV-2 pandemic must account for the potential role of political ideology. The pandemic was highly politicized [32] when it reached the United States (U.S.). For example, viewership of conservative cable media was associated with decreased propensity to stay home and physically distance from others [33]. This may reflect differences in how liberal and conservative ideologies moralize issues [34] as more liberal individuals were more likely to view appeals to protect public health as moral arguments [35]. Recent work has shown that partisanship was associated with early responses to the pandemic, such that Republicans and conservatives were less likely to engage in preventive health behaviors or report COVID-19 related attitudes conducive to health behavior engagement [36, 37]. Moreover, the role of partisanship has grown over time [38]. Thus, there is evidence that individual differences in political beliefs contribute to divergent responses to the pandemic, and exploration of political beliefs is especially important in the earliest months of the pandemic.

To test our hypotheses that compassion and empathy would be associated with more prosocial behaviors and attitudes, respectively, concerning the SARS-CoV-2 pandemic, we fielded a survey to over 5,000 participants every two weeks during a 14-week period starting in late March 2020, shortly after the first U.S. statewide shelter-in-place order was implemented [39]. This paper reports our findings.

## Method

### Design

From March 22 to June 15, 2020, data were collected from seven cohorts at two-week intervals. Participants were eligible for the 15-to-20-minute research study titled “Thoughts and Feelings about COVID-19”, if they were at least 18 years of age, were fluent in English, and resided in the U.S. Participants were recruited from two convenience samples via Amazon Mechanical Turk (MTurk) and Qualtrics Online Panels (Qualtrics), with a three-day window for survey completion. Participants consented to participate the study by clicking “agree” on the study information page. Quotas were set to recruit roughly equal numbers of men and women to account for potential gender differences in self-reported compassion and empathy [40]. Cohorts 1 and 2 were initially restricted to MTurk respondents from California and Florida, and additional funding was obtained to open recruitment to the entire U.S. starting at Cohort 3, as well as open recruitment to Qualtrics starting at Cohort 4. MTurk respondents were eligible if they had at least a 95% approval rate and were compensated $5 for completing the survey. Qualtrics recruited and compensated respondents in collaboration with various market research platforms. The number of respondents who did not complete the survey are shown in S1 Table in S1 File. In addition, neither the five respondents who reported their sex as “other” were included in the study (as this sample was too small to detect any differences between groups), nor were the 147 respondents who declined to state their income (as income was a covariate in all analyses). A total of 5,533 participants were included in data analyses. At the Cohort 3 fielding and again at the Cohort 6 fielding, survey questions were added to capture responses to the evolving pandemic and are described in the next section. The full survey instrument is available upon request.

### Measures

#### Demographics

After consenting to the terms of the study, all participants answered demographics questions about age, sex (female vs. male), race (Asian, Black/African American, Other, and White as the reference group), ethnicity (Hispanic/Latino, non-Hispanic/Latino), degree of educational attainment (less than four-year college degree, more than four-year college degree, and four-year college degree as the reference group), and income ($0-$49,999, $50,000-$99,999, $100,000-$149,999, $150,000-$199,999). We aggregated the race categories of American Indian or Alaska Native, Native Hawaiian or Other Pacific Islander, and More than one race with the Other group as each group was small and underpowered to detect differences between groups.

#### Compassion and empathy

To assess dispositional compassion and empathy, participants completed the previously validated 5-item Santa Clara Brief Compassion Scale [41], and the 16-item Toronto Empathy Questionnaire [8].

#### Political beliefs

Political ideology was assessed by asking how participants usually think about their political beliefs on a 7-point Likert scale from 1 (*Strongly conservative*) through 4 (*Neither*) to 7 (*Strongly liberal*).

#### Prosocial health behaviors

*Physical distancing (Cohorts 1–7)*. Participants reported whether, in the last two weeks, they engaged in behaviors “which a person might do in response to coronavirus (COVID-19)”. Participants reported whether they voluntarily stayed home to “prevent others from getting sick” (yes, no). They answered two questions about physical distancing, which asked how many hours per day, on average, did they spend “staying home when you would normally have gone out?” and “keeping a distance from other people, more than you normally would?”. The drop-down menu ranged from zero to 11+ hours, then response options were collapsed into six categories (hours: 0, 1–2, 3–5, 6–8, 9–10, 11–12).

*Mask wearing (Cohort 6–7 only)*. Starting at Cohort 6, participants also rated on a 5-point Likert scale from *never* to *always* the following item about the last two weeks, “When I was out in public, I wore a face mask or covering”.

#### Ability to help others (Cohort 3–7 only)

Starting at Cohort 3, participants reported their ability to help others, “I feel in a position to help people who may be negatively affected by coronavirus (COVID-19)”, using a 5-point Likert scale from 1 (*Strongly disagree*) to 5 (*Strongly agree*).

#### Prosocial attitudes

*Views about others’ reactions to COVID-19*. Participants rated their agreement with two statements designed to assess prosocial attitudes, “I can understand why people are afraid of coronavirus (COVID19)” and “People are overreacting to coronavirus (COVID-19; reverse scores indicating more prosocial attitude)”, using the 5-point Likert scale of disagreement vs. agreement.

*Understanding of the benefit of sheltering-place (Cohort 3–7 only)*. Starting at Cohort 3, participants used the same 5-point scale of disagreement vs. agreement to report their understanding of the relationship between sheltering-in-place and protecting others’ health, rating the statement, “When I stay home, I am preventing the spread of coronavirus (COVID-19)”.

#### Other covariates

To assess potential biases in self-presentation, participants completed the 10-item Marlow-Crown Social Desirability Scale [42]. To assess pandemic-related factors, participants were asked “Are you currently required by your employer to work remotely?” (response options: yes, no, I’m not currently employed, and Prefer not to answer, which was collapsed with the “unemployed” category). Participants also answered the question, “Have you been diagnosed with coronavirus (COVID-19)?” (yes, no). If participants indicated they had children, they reported whether their “childcare responsibilities increased due to coronavirus (COVID-19; yes, no)”.

### Statistical analyses

Assessments every two weeks were designed to capture potential differences in responses during the first four months of the pandemic. However, prior to conducting our analyses, we tested whether there was significant variability in any of the seven outcome variables across the seven cohorts, and the intraclass correlation coefficients (intraclass correlations < 0) indicated no variability based on cohort. Therefore, data from all seven cohorts were combined to increase statistical power. Participant characteristics are described stratified separately by compassion and empathy (median split) in Table 1. It was determined *a priori* to examine compassion in relation to prosocial health behaviors separately from empathy in relation to prosocial attitudes. In the first model, potential confounders of age, sex, race, ethnicity, education, income, employment, social desirability, personal diagnosis of COVID-19, increased childcare responsibilities, cohort number, and sample source (MTurk or Qualtrics) were added to the models. To examine the influence of political ideology, a second model was fit including all Model 1 covariates and political ideology. To improve interpretability, compassion, empathy, and all continuous covariates are reported using standardized beta coefficients. All available data were used for each outcome by leveraging complete case analysis implemented by logistic and linear regression for binary and continuous outcomes, respectively. Analyses were conducted using IBM SPSS Statistics 27.

**Table 1:** Participant characteristics (March 2020 through July 2020).

Characteristic	*N (%)* or *mean (SD)*
Age, *mean (SD)*	46.86 (15.81)
Female sex, *n (%)*	2806 (51%)
Race/ethnicity, *n (%)*	
Asian	345 (6%)
Black	513 (9%)
White	4403 (80%)
Other Race	272 (5%)
Hispanic/Latino Ethnicity, *n (%)*	573 (10%)
Education, *n (%)*	
Less than college	2139 (39%)
College degree	1961 (35%)
More than college	1433 (26%)
Employment, *n (%)*	
Non-remote work	1464 (27%)
Remote work	2219 (40%)
Unemployed	1850 (33%)
Income, *n (%)*	
$0-$49,999	1978 (36%)
$50,000-$99,999	2126 (38%)
$100,000-$149,999	896 (16%)
> $150,000	533 (10%)
Increased childcare (Yes), *n (%)*	983 (18%)
COVID-19 diagnosis (Yes), *n (%)*	86 (2%)
Social desirability, *mean (SD)*	5.17 (2.38)
Political ideology, *mean (SD)*	4.30 (1.90)

**Fig 1:**
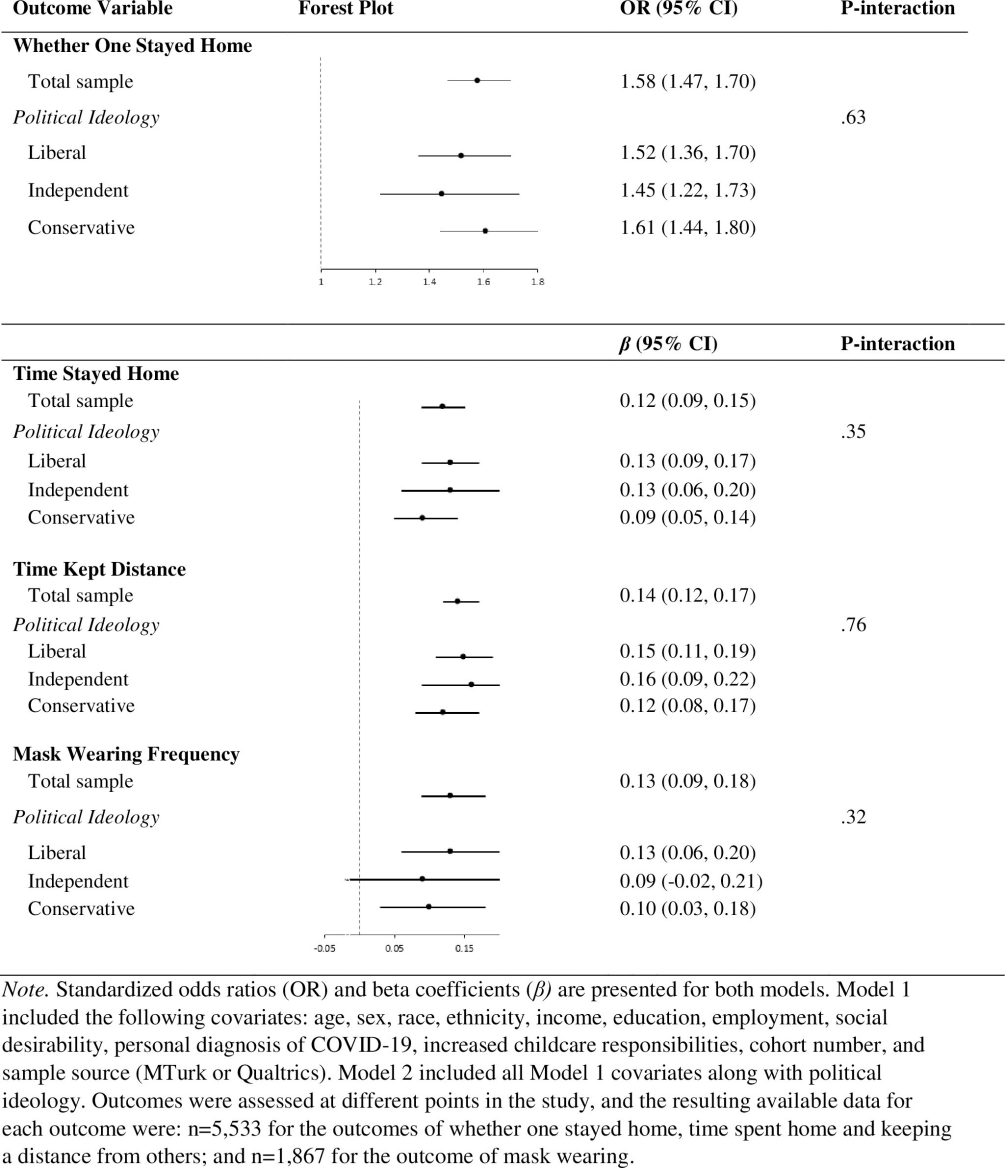
Depicts the forest plots for the associations (standardized odds ratios or beta coefficients) between compassion and three health behaviors: Whether one stayed home, time stayed home, time keeping distance from others, and mask wearing frequency. The first forest plot for each health behavior outcome is for the total sample, and the following three forest plots are for the sub-groups of liberal-leaning, independent, and conservative-leaning participants. The figure also depicts the 95% confidence intervals and the p-value for the interaction term between compassion and the continuous variable of political ideology.

**Fig 2:**
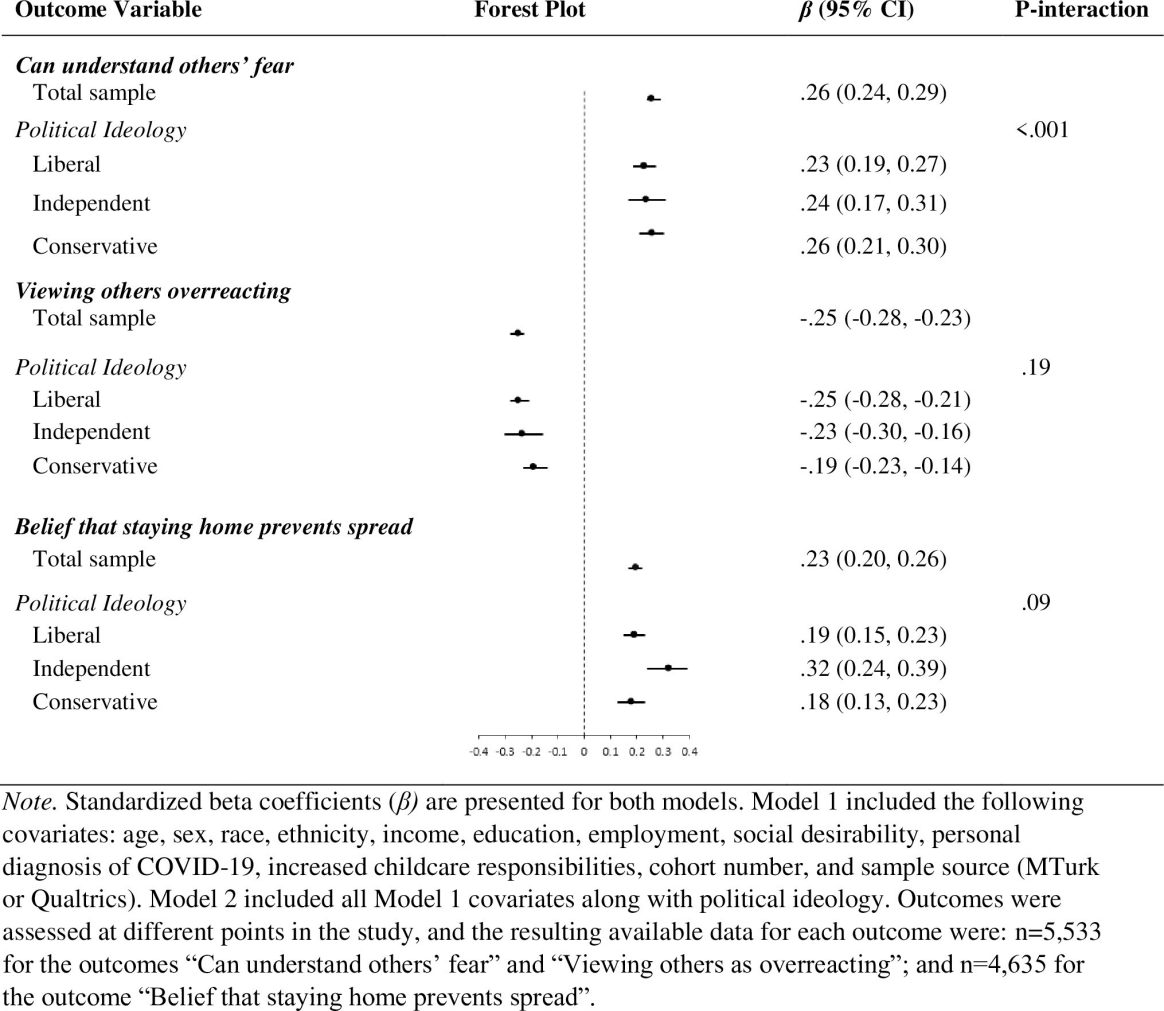
Depicts the forest plots for the associations (standardized beta coefficients) between empathy and three prosocial attitudes: Can understand others’ fear, viewing others as overreacting, and belief that staying home prevents the spread of COVID-19. The first forest plot for each attitude outcome is for the total sample, and the following three forest plots are for the sub-groups of liberal-leaning, independent, and conservative-leaning participants. The figure also depicts the 95% confidence intervals and the p-value for the interaction term between empathy and political ideology.

We also assessed political ideology as a potential effect modifier of associations of compassion or empathy with prosocial behaviors or attitudes, using two approaches. First, in fully adjusted Model 3, we added a multiplicative interaction term for political ideology and compassion or empathy and report the resulting *p*-value for the interaction. Second, we repeated all models stratified by three categories of political ideology: liberal, independent, and conservative and visualized the results in Figs 1 and 2.

## Results

Participant characteristics are displayed in Table 1. A higher proportion of women, liberal-leaning participants, and those reporting higher social desirability were at or above the median of compassion and empathy (S2 Table in S1 File). Descriptive statistics and zero-order correlations for compassion, empathy, political ideology, and the outcome variables are displayed in S3 Table in S1 File.

### Compassion and prosocial health behaviors

Each standard deviation increment in compassion was associated with 55% higher odds of having stayed home to protect others from getting sick, (odds ratio: 1.55, *p* < .001; Table 2), accounting for all covariates including political ideology. Each standard deviation increment in compassion was associated with more time spent staying home than usual (*β* = .12; 95% CI = 0.09,0.14), more time spent keeping a distance from others than usual (*β* = .14; 95% CI = 0.11,0.16), and more frequent mask wearing in public (*β* = .11; 95% CI = 0.06,0.15; all *p*s < .001)—there was no evidence of moderation by political ideology (all *p*-interactions ≥ 0.32; Fig 1; S17-S27 Outputs in S1 File display models by political ideology group). We examined prosocial efficacy as a separate outcome and found that each standard deviation increment in compassion was associated with more perceived ability to help others negatively affected by the pandemic (*β* = .33; 95% CI = 0.30, 0.36; *p* < .001)—the association was not modified by political ideology (*p-*interaction = .55; see S3-S6 Tables in S1 File for results for covariates included in these analyses). The full models with all covariates included are displayed in S4, S5 and S8, S9 Tables in S1 File. Because there was a main effect of sample source in the model predicting time spent staying home, S6, S7 Tables in S1 File display the results of the model when only including MTurk or Qualtrics participants.

**Table 2:** Standardized odds ratios and regression coefficients for dispositional compassion and empathy in relation to health behaviors and prosocial attitudes, in models with and without political ideology.

Exposure Variable	Outcome Variable	Model 1		Model 2	
	*Health Behavior Engagement*	*Odds ratio* (95% CI)	*P*	*Odds ratio* (95% CI)	*P*
Compassion	Whether one stayed home for others	1.58 (1.47, 1.70)	< .001	1.55 (1.44, 1.66)	< .001
	*Health Behavior Frequency*	*β* (95% CI)	P	*β* (95% CI)	P
Compassion	Time spent staying home	0.12 (0.09, 0.15)	< .001	0.11 (0.09, 0.14)	< .001
Compassion	Time keeping distance from others	0.14 (0.12, 0.17)	< .001	0.14 (0.11, 0.16)	< .001
Compassion	Frequency of mask wearing in public	0.13 (0.09, 0.18)	< .001	0.11 (0.06, 0.15)	< .001
	*Prosocial Attitudes*				
Empathy	Can understand others’ fear	-0.26 (0.23, 0.29)	< .001	0.23 (0.20, 0.26)	< .001
Empathy	Viewing others as overreacting	-0.25 (-0.28, -0.23)	< .001	-0.19 (-0.22, -0.17)	< .001
Empathy	Belief that staying home prevents spread	-0.23 (0.20, 0.26)	< .001	-0.23 (0.20, 0.25)	< .001

*Note*. Standardized odds ratios and beta coefficients (*β)* are presented for both models. Model 1 included the following covariates: age, sex, race, ethnicity, gender, income, education, employment, social desirability, personal diagnosis of COVID-19, increased childcare responsibilities, cohort number, and sample source (MTurk or Qualtrics). Model 2 included all Model 1 covariates along with political ideology. Outcomes were assessed at different points in the study, and the resulting available data for each outcome were: n = 5,533 for the outcomes of whether one stayed home, time spent home and keeping a distance from others; n = 1,867 for the outcome of mask wearing; n = 5,533 for the outcomes “Can understand others’ fear” and “Viewing others as overreacting”; and n = 4,635 for the outcome “Belief that staying home prevents spread”.

### Empathy and prosocial attitudes

In general, empathy was more strongly related to prosocial attitudes than compassion was to prosocial behaviors, as indicated by the larger standardized beta coefficients and largely non-overlapping 95% CIs. Each standard deviation increment in empathy was associated with less endorsement of the view that others were overreacting to COVID-19 (*β* = -.19; 95% CI = -0.22, 0.17), and greater belief that staying home prevents the spread of the virus (*β* = .20; 95% CI = 0.16, 0.22). Greater empathy was more strongly associated with understanding of why people are afraid of COVID-19 among people with a conservative versus liberal political ideology (*p*-interaction < 0.001; Fig 2): standardized beta coefficients (95% CIs) were 0.23 (0.19, 0.27), 0.24 (0.17, 0.31), and 0.26 (0.21, 0.30) for people with liberal, independent, and conservative political ideologies, respectively. No other significant moderation by political ideology was observed (see S10, S13, S14 Tables in S1 File for results for covariates included in these analyses; S28-S36 Outputs in S1 File display models by political ideology group). Because there was a main effect of sample source in the models predicting understanding of others’ fear and the belief that staying home prevents the spread, S11, S12 and S15, S16 Tables in S1 File display the results of the models when only including MTurk or Qualtrics participants.

### Exploratory analyses of compassion, empathy, and political ideology

Because of the early politicized nature of pandemic responses in the U.S, we accounted for political ideology when examining the relationships between compassion and prosocial behaviors, and between empathy and prosocial attitudes. To examine the statistical effects of political ideology further, we conducted exploratory follow-up correlation analyses. Specifically, we found that when holding empathy constant among participants scoring at the median, 25^th^ and 75^th^ percentiles, political ideology still played a role in prosocial attitudes (S37 Table in S1 File). These follow-up analyses are consistent with the statistically significant main effects for political ideology found in regressions predicting prosocial attitudes.

In contrast, we found that when holding compassion constant among participants scoring at the median, 25^th^ and 75^th^ percentiles, political ideology only played a role in two of the four behavior variables (whether one stayed home for others and frequency of mask wearing in public; S39 Table in S1 File). These follow-up up analyses are consistent with the finding that effect sizes for political ideology were larger in regressions predicting the prosocial behaviors that may have been more politically motivated (whether one stayed home for others, frequency of mask wearing in public) relative to those assessing degree of behavior (time spent staying home or keeping a distance more than one normally would).

## Discussion

This investigation of over 5,000 participants during the first four months of the U.S. outbreak of SARS-CoV-2 is among the first to produce empirical evidence that dispositional compassion is associated with health behaviors during a pandemic. Participants with more compassion were more likely to have stayed home to protect others from getting sick in the past two weeks, and spent more time staying home, keeping distance from others, and wearing masks in public. Importantly, these effects were statistically independent of political ideology. Future work should test causal directions of these relationships to determine if improving compassion can increase health behaviors during a pandemic.

In previous research, state compassion was increased by experimental emotion procedures [12, 16]. Our findings, however, suggest testing whether increasing the overall tendency to have compassion for others in daily life has benefits for health behaviors. A randomized control trial found empathy training increases patients’ reports of resident physicians’ compassion [43]. Additionally, self-reports of dispositional compassion can increase as a result of compassion training, such as Compassion Cultivation Training, which includes education about having a compassionate mindset toward others [44]. However, compassion training is often opt-in. Large-scale benefits from compassion may require incentivization and mandates at organizational and institutional levels [45] as well as changes in public messaging and social norms [46]. One feasible approach could be to test whether micro-interventions, such as fostering positive social connections and cultivating co-experiences of pleasant emotional states could increase compassion and empathy in daily life [47].

In the current study, participants with more dispositional empathy also more strongly endorsed prosocial attitudes. Empathy was associated with more understanding for others’ fears, for instance, and this association was stronger among more conservative-leaning individuals. Any intervention tailored to political orientation, however, should consider ideological differences in motivated reasoning, as it may be necessary, for instance, to leverage moral values [34] underpinning conservative ideology (e.g., purity, loyalty), which might otherwise be at odds with following public health directives [35]. Relatedly, calls for compassion and empathy among more conservative-leaning individuals should be compatible with the ways in which individuals with such beliefs may express prosocial concerns, such as by focusing more on protecting small businesses during the pandemic [13], or more generally, by having more empathy for specific and known individuals (e.g., family members, friends) compared to strangers, or humanity [48]. Interventions could also consider seeking to increase empathy toward members of an outgroup, whether based on political ideology or other factors [49], however such approaches should avoid making political ideology salient (e.g., highlighting polarized responses to COVID-19) in ways that could inadvertently encourage tribalism. Given our finding that overall, understanding for others was moderately correlated with the degree of viewing others as overreacting, it may be possible to increase individuals’ empathy for others’ reactions to COVID-19 regardless of whether they, themselves, think those reactions are warranted. For instance, more conservative individuals may have reasons to view others as overreacting but still wear a mask out of concern for members of their families or communities.

More work is needed to address the limitations of this investigation. Most participants were recruited from MTurk, where respondents tend to be more representative of the general population than college student convenience samples, but are also more liberal-leaning than the general population. Recent work has shown that studies recruiting participants from such online convenience platforms can overestimate prosocial behaviors [50]. However, the mean score for dispositional empathy in our sample was similar to the mean for a related construct, empathic concern [51, 52], as assessed in a nationally representative sample of over 2,000 adults in the U.S. from the 2008–2009 American National Election Studies panel data [49] (see S39 Table in S1 File). Our research aim was not to gage prevalence or degree of prosocial behaviors and attitudes, but to examine the extent to which empathy and compassion were associated with such behaviors and attitudes. Nonetheless, drawing from an MTurk sample was also limited in that participants tend to be younger and more educated than the general population, and thus we also recruited participants from Qualtrics Panels who tend to be more representative of the general population [53, 54]. However, there may be other characteristics of these two convenience samples that limit inferences that can made about the general population in the U.S., and more research is needed to determine whether dispositional compassion and empathy broadly predict prosocial health behaviors and attitudes during a pandemic. The majority of cohorts were predominantly White and research is needed to determine whether findings replicate in samples with more racial and ethnic diversity as well as in samples with lower socioeconomic status, for which sheltering-in-place may be more difficult and/or detrimental [55].

Our interpretation of our findings is that more compassionate individuals show more prosocial behaviors because of their concern for others. However, a possible alternative explanation is that these individuals’ prosocial orientation helps them understand the link between social contact and increased risk of disease transmission, which can then motivate preventive health behaviors. Disentangling prosocial from self-interested motivations may require experimental designs [12] but such distinctions are not necessary for ultimately changing behaviors to improve public health. Importantly, we did not find consistent effects of social desirability in relating to health behaviors.

In conclusion, we found evidence that during the SARS-CoV-2 pandemic, compassion was related to engaging in more health behaviors of benefit to public health, and empathy was related to more prosocial attitudes toward others. Cultivating compassion and empathy for others may be especially important for bridging the political divide in the U.S. concerning people’s responses to a pandemic.

## Supporting information

S1 File (DOCX)

S2 File (SAV)
