# Supplementary material for: Association of compassion and empathy with prosocial health behaviors and attitudes in a pandemic
Source: PLoS One. 2022 Jul 22;17(7):e0271829. doi: 10.1371/journal.pone.0271829 (PMC9307157; doi:10.1371/journal.pone.0271829)
Supplement: S1 File — (DOCX) [file pone.0271829.s001.docx]

**S1 Table 1.** Number of MTurk and Qualtrics Respondents Who Enrolled, Were Excluded based on Quality Checks, and Completed the Survey, by Cohort and Recruitment

|  |  |  | Number of Respondents | | | | | | | |
| --- | --- | --- | --- | --- | --- | --- | --- | --- | --- | --- |
| Cohort | Recruitment | Date |  | Enrolled | |  | Excluded | | With complete data | |
| 1 | MTurk | March 22, 2020 |  |  | 514 |  |  | 1 was not within age range of <91 years | 513 |  |
| 2 | MTurk | April 6, 2020 |  |  | 400 |  |  | 5 withdrew | 395 |  |
| 3 | MTurk | April 20, 2020 |  |  | 646 |  |  | 5 withdrew | 641 |  |
| 4 | MTurk | May 4, 2020 |  |  | 588 |  |  | 8 withdrew | 580 |  |
| 4 | Qualtrics | May 4, 2020 |  |  | 658 |  |  | 95 withdrew  49 did not pass Qualtrics quality check | 514 |  |
| 5 | MTurk | May 18, 2020 |  |  | 590 |  |  | 4 withdrew  1 was not within age range of <91 years | 585 |  |
| 5 | Qualtrics | May 18, 2020 |  |  | 1075 |  |  | 475 withdrew  78 did not pass Qualtrics quality check | 522 |  |
| 6 | MTurk | June 1, 2020 |  |  | 311 |  |  | 3 withdrew  7 had technical error resulting in missing face mask question | 301 |  |
| 6 | Qualtrics | June 1, 2020 |  |  | 701 |  |  | 84 withdrew  115 did not pass Qualtrics quality check  1 was not within age range of <91 years | 501 |  |
| 7 | MTurk | June 1, 2020 |  |  | 521 |  |  | 6 withdrew | 515 |  |
| 7 | Qualtrics | June 15, 2020 |  |  | 1417 |  |  | 754 withdrew  44 did not pass Qualtrics quality check  1 was not within age range of <91 years | 618 |  |
|  |  |  |  |  |  |  |  | Respondents who completed the survey: | 5685 |  |

*Note.* Respondents who exited the survey before completing the first question concerning the SARS-CoV-2 pandemic (presented after the demographic questions) were not counted as enrolled in the study.

| **S2 Table.** Participant Characteristics by Dispositional Compassion and Empathy (March 2020 through July 2020) | | | | | | |
| --- | --- | --- | --- | --- | --- | --- |
|  | Compassion | | | Empathy | | |
| Characteristic | below median | at or above median | *P* Value | below median | at or above median | *P* Value |
| Age*, mean (SD)* | 47.64 (16.24) | 46.50 (15.62) | .007 | 46.48 (16.18) | 47.56 (15.68) | .007 |
| Female sex*, n (%)* | 1140 (42.4%) | 1748 (58.5%) | <.001 | 1074 (39.4%) | 1814 (61.4%) | <.001 |
| Race/ethnicity*, n (%)* |  |  | <.001 |  |  | <.001 |
| Asian | 193 (7.2%) | 165 (5.5%) |  | 211 (7.7%) | 147 (5.0%) |  |
| Black | 184 (6.8%) | 339 (11.3%) |  | 222 (8.1%) | 301 (10.2%) |  |
| White | 2184 (81.1%) | 2340 (78.3%) |  | 2146 (78.6%) | 2378 (80.5%) |  |
| Other Race | 133 (4.9%) | 146 (4.9%) |  | 152 (5.6%) | 127 (4.3%) |  |
| Hispanic/Latino Ethnicity*, n (%)* | 253 (9.4%) | 335 (11.2%) | .03 | 314 (11.5%) | 274 (9.3%) | .03 |
| Education*, n (%)* |  |  | .86 |  |  | .03 |
| Less than college | 755 (38.5%) | 1140 (38.1%) |  | 996 (36.5%) | 1180 (40.0%) |  |
| College degree | 959 (35.6%) | 1055 (35.3%) |  | 998 (36.5%) | 1016 (34.4%) |  |
| More than college | 699 (25.9%) | 795 (26.6%) |  | 737 (27.9%) | 757 (25.6%) |  |
| Employment*, n (%)* |  |  | <.001 |  |  | .002 |
| Non-remote work | 755 (28.0%) | 745 (24.9%) |  | 773 (28.3%) | 727 (24.6%) |  |
| Remote work | 979 (36.3%) | 1267 (42.4%) |  | 1077 (39.4%) | 1169 (39.6%) |  |
| Unemployed | 960 (35.6%) | 978 (32.7%) |  | 881 (32.3%) | 1057 (35.8%) |  |
| Income*, n (%)* |  |  | .05 |  |  | .03 |
| $0-$49,999 | 911 (35.0%) | 1068 (36.4%) |  | 909 (34.3%) | 1070 (37.1%) |  |
| $50,000-$99,999 | 977 (37.5%) | 1152 (39.3%) |  | 1016 (38.3%) | 1113 (28.6%) |  |
| $100,000-$149,999 | 453 (17.4%) | 443 (15.1%) |  | 459 (17.3%) | 437 (15.2%) |  |
| > $150,000 | 265 (10.2%) | 268 (9.1%) |  | 270 (10.2%) | 263 (9.1%) |  |
| Increased childcare (Yes)*, n (%)* | 408 (15.1%) | 580 (19.4%) | <.001 | 489 (17.9%) | 499 (16.9%) | <.001 |
| COVID-19 diagnosis (Yes)*, n (%)* | 31 (1.2%) | 55 (1.8%) | .03 | 78 (2.9%) | 8 (0.3%) | .03 |
| Social desirability*, mean (SD)* | 4.90 (2.37) | 5.46 (2.36) | <.001 | 4.92 (2.29) | 5.45 (2.43) | <.001 |
| Political ideology*, mean (SD)* | 4.09 (1.82) | 4.47 (1.94) | <.001 | 4.00 (1.81) | 4.57 (1.93) | <.001 |

*Note.* The median of compassion scores was 5.00. The median of empathy scores was 2.80

**S3 Table.** Descriptive Statistics and Correlations among Compassion, Empathy, Political Ideology, and Prosocial Health Behaviors and Attitudes

|  |  | |  | | Correlations | | | | | | | | | |
| --- | --- | --- | --- | --- | --- | --- | --- | --- | --- | --- | --- | --- | --- | --- |
| Variable | N | M | SD | Range | 1 | 2 | 3 | 4 | 5 | 6 | 7 | 8 | 9 | 10 |
| 1. Compassion | 5333 | 4.90 | 1.26 | 1.00 - 7.00 | 1 | .64*** | .09*** | .16*** | .16*** | .16*** | .34*** | .15*** | -.18*** | .19*** |
| 2. Empathy | 5333 | 2.77 | 0.59 | 0.00 - 4.00 |  | 1 | .16*** | .11*** | .12*** | .15*** | .18*** | .26*** | -.28*** | .23*** |
| 3. Political  ideology | 5333 | 4.30 | 1.90 | 1.00 - 7.00 |  |  | 1 | .07*** | .08*** | .22*** | 0.01 | .25*** | -.40*** | .25*** |
| 4. Time home | 5333 | 3.43 | 2.05 | 0.00 - 6.00 |  |  |  | 1 | .67*** | .26*** | .11*** | .12*** | -.17*** | .18*** |
| 5. Time keeping  distance | 5333 | 3.57 | 2.14 | 0.00 - 6.00 |  |  |  |  | 1 | .25*** | .11*** | .14*** | -.19**** | .19*** |
| 6. Mask wearing  frequency | 1867 | 4.20 | 1.18 | 1.00 - 5.00 |  |  |  |  |  | 1 | .16*** | .29*** | -.44*** | .43*** |
| 7. Ability to help others | 4635 | 3.14 | 1.08 | 1.00 - 5.00 |  |  |  |  |  |  | 1 | .07*** | -.08*** | .21*** |
| 8. “Understand why others afraid” | 5333 | 4.48 | 0.89 | 1.00 - 5.00 |  |  |  |  |  |  |  | 1 | -.45*** | .39*** |
| 9. “People are overreacting” | 5333 | 2.12 | 1.34 | 1.00 - 5.00 |  |  |  |  |  |  |  |  | 1 | -.48*** |
| 10. Efficacy of sheltering in place | 4635 | 4.29 | 0.97 | 1.00 - 5.00 |  |  |  |  |  |  |  |  |  | 1 |

*Note. *** p < .001.*

**S4 Table.** Predicting Whether One Stayed Home for Others with Multinomial Logistic Regression

|  | Model 1 | | |  | | Model 2 | | |  | | Model 3 | | |
| --- | --- | --- | --- | --- | --- | --- | --- | --- | --- | --- | --- | --- | --- |
| Predictor | *Odds ratio*  [95% CI] | *SE* | *p* |  | *Odds ratio*  [95% CI] | | *SE* | *p* |  | *Odds ratio*  [95% CI] | | *SE* | *p* |
| Age | 0.96 [0.88, 1.05] | 0.05 | .37 |  | 1.01 [0.92, 1.11] | | 0.05 | .82 |  | 1.01 [0.92, 1.11] | | 0.05 | .81 |
| Female | 1.17 [1.01, 1.35] | 0.07 | .03 |  | 1.16 [1.00, 1.34] | | 0.07 | .04 |  | 1.16 [1.00, 1.34] | | 0.07 | .04 |
| *Race* |  |  |  |  |  | |  |  |  |  | |  |  |
| Asian | 1.57 [1.12, 2.22] | 0.17 | .009 |  | 1.48 [1.05, 2.09] | | 0.18 | .03 |  | 1.48 [1.05, 2.09] | | 0.18 | .03 |
| Black | 0.86 [0.68, 1.09] | 0.12 | .22 |  | 0.76 [0.60, 0.97] | | 0.12 | .03 |  | 0.76 [0.60, 0.97] | | 0.12 | .03 |
| Other Race | 1.10 [0.77, 1.58] | 0.18 | .60 |  | 1.03 [0.72, 1.48] | | 0.18 | .85 |  | 1.03 [0.72, 1.48] | | 0.18 | .86 |
| Hispanic/Latino | 1.39 [1.07, 1.81] | 0.13 | .01 |  | 1.38 [1.06, 1.80] | | 0.14 | .02 |  | 1.38 [1.06, 1.79] | | 0.14 | .02 |
| *Education* |  |  |  |  |  | |  |  |  |  | |  |  |
| Less than college | 0.91 [0.76, 1.07] | 0.09 | .25 |  | 0.94 [0.80, 1.12] | | 0.09 | .51 |  | 0.95 [0.80, 1.12] | | 0.09 | .52 |
| More than college | 1.02 [0.85, 1.23] | 0.10 | .83 |  | 0.99 [0.82, 1.20] | | 0.10 | .94 |  | 0.99 [0.82, 1.20] | | 0.10 | .96 |
| *Employment* |  |  |  |  |  | |  |  |  |  | |  |  |
| Remote work | 2.78 [2.34, 3.32] | 0.09 | <.001 |  | 2.77 [2.32, 3.31] | | 0.09 | <.001 |  | 2.77 [2.32, 3.31] | | 0.09 | <.001 |
| Unemployed | 2.59 [2.17, 3.09] | 0.09 | <.001 |  | 2.43 [2.03, 2.91] | | 0.09 | <.001 |  | 2.44 [2.04, 2.91] | | 0.09 | <.001 |
| Income | 0.98 [0.90, 1.06] | 0.04 | .59 |  | 0.99 [0.91, 1.07] | | 0.04 | .80 |  | 0.99 [0.91, 1.07] | | 0.04 | .80 |
| Social desirability | 0.96 [0.89, 1.03] | 0.04 | .27 |  | 0.98 [0.91, 1.06] | | 0.04 | .65 |  | 0.98 [0.91, 1.06] | | 0.04 | .65 |
| Diagnosed with COVID | 1.77 [0.83, 3.79] | 0.39 | .14 |  | 2.07 [0.97, 4.42] | | 0.39 | .06 |  | 2.06 [0.96, 4.42] | | 0.39 | .06 |
| Increased childcare | 1.23 [1.00, 1.52] | 0.11 | .05 |  | 1.32 [1.06, 1.63] | | 0.11 | .01 |  | 1.32 [1.06, 1.63] | | 0.11 | .01 |
| Cohort | 0.85 [0.81, 0.89] | 0.02 | <.001 |  | 0.85 [0.81, 0.88] | | 0.02 | <.001 |  | 0.85 [0.81, 0.88] | | 0.02 | <.001 |
| Qualtrics vs. MTurk | 0.93 [0.77, 1.13] | 0.10 | .47 |  | 1.01 [0.83, 1.22] | | 0.10 | .94 |  | 1.01 [0.83, 1.22] | | 0.10 | .94 |
| Compassion | 1.58 [1.47, 1.70] | 0.04 | <.001 |  | 1.55 [1.44, 1.66] | | 0.04 | <.001 |  | 1.54 [1.43, 1.66] | | 0.04 | <.001 |
| Political ideology |  |  |  |  | 1.4 [1.29, 1.50] | | 0.04 | <.001 |  | 1.39 [1.29, 1.50] | | 0.04 | <.001 |
| Compassion X Political ideology |  |  |  |  |  | |  |  |  | 0.98 [0.92, 1.05] | | 0.04 | .63 |

*Note.* Age, social desirability, compassion, and political ideology scores were standardized. *N* = 5533. The Hosmer-Lemeshow statistic indicated that Model 1 fit the data, *Х*^2^(8)=6.92, p = .55, Model 2 fit the data, *Х*^2^(8)=7.44, p = .49, and Model 3 fit the data, *Х*^2^(8)=7.19, p = .52.

**S5 Table.** Predicting Time Spent Home with Multiple Regression

|  | Model 1 | | |  | Model 2 | | |  | Model 3 | | |
| --- | --- | --- | --- | --- | --- | --- | --- | --- | --- | --- | --- |
| Predictor | *Β* [95% CI] | *SE* | *p* |  | *Β* [95% CI] | *SE* | *p* |  | *Β* [95% CI] | *SE* | *p* |
| Age | -0.01 [-0.04, 0.03] | 0.02 | .87 |  | 0.01 [-0.03, 0.04] | 0.02 | .76 |  | 0.01 [-0.03, 0.04] | 0.02 | .78 |
| Female | 0.05 [0.04, 0.14] | 0.03 | .001 |  | 0.05 [0.04, 0.14] | 0.03 | .001 |  | 0.05 [0.04, 0.14] | 0.03 | .001 |
| *Race* |  |  |  |  |  |  |  |  |  |  |  |
| Asian | 0.07 [0.16, 0.38] | 0.06 | <.001 |  | 0.06 [0.15, 0.37] | 0.06 | <.001 |  | 0.06 [0.15, 0.37] | 0.06 | <.001 |
| Black | 0.06 [0.13, 0.31] | 0.05 | <.001 |  | 0.06 [0.12, 0.30] | 0.05 | <.001 |  | 0.06 [0.12, 0.30] | 0.05 | <.001 |
| Other Race | 0.02 [-0.03, 0.21] | 0.06 | .16 |  | 0.02 [-0.04, 0.20] | 0.06 | .21 |  | 0.02 [-0.04, 0.20] | 0.06 | .21 |
| Hispanic/Latino | 0.08 [0.18, 0.36] | 0.05 | <.001 |  | 0.08 [0.18, 0.36] | 0.05 | <.001 |  | 0.08 [0.18, 0.36] | 0.05 | <.001 |
| *Education* |  |  |  |  |  |  |  |  |  |  |  |
| Less than college | 0.01 [-0.04, 0.09] | 0.03 | .40 |  | 0.02 [-0.03, 0.10] | 0.03 | 0.29 |  | 0.02 [-0.03, 0.09] | 0.03 | .29 |
| More than college | 0.01 [-0.04, 0.09] | 0.03 | .48 |  | 0.01 [-0.05, 0.09] | 0.03 | 0.58 |  | 0.01 [-0.05, 0.09] | 0.03 | .59 |
| *Employment* |  |  |  |  |  |  |  |  |  |  |  |
| Remote | 0.17 [0.28, 0.41] | 0.03 | <.001 |  | 0.17 [0.28, 0.41] | 0.03 | <.001 |  | 0.17 [0.28, 0.41] | 0.03 | <.001 |
| Unemployed | 0.02 [-0.02, 0.12] | 0.03 | .13 |  | 0.02 [-0.03, 0.11] | 0.03 | .25 |  | 0.02 [-0.03, 0.11] | 0.03 | .25 |
| Income | 0.04 [0.01, 0.07] | 0.02 | .01 |  | 0.04 [0.01, 0.07] | 0.02 | .009 |  | 0.04 [0.01, 0.07] | 0.02 | .008 |
| Social desirability | 0.05 [0.02, 0.08] | 0.01 | <.001 |  | 0.05 [0.03, 0.08] | 0.01 | <.001 |  | 0.05 [0.03, 0.08] | 0.01 | <.001 |
| COVID-19 diagnosis | -0.05 [-0.63, -0.21] | 0.11 | <.001 |  | -0.05 [-0.59, -0.18] | 0.11 | <.001 |  | -0.05 [-0.59, -0.17] | 0.11 | <.001 |
| Increased childcare | 0.06 [0.08, 0.22] | 0.04 | <.001 |  | 0.06 [0.09, 0.23] | 0.04 | <.001 |  | 0.06 [0.09, 0.23] | 0.04 | <.001 |
| Cohort | -0.08 [-0.06, -0.03] | 0.01 | <.001 |  | -0.08 [-0.06, -0.03] | 0.01 | <.001 |  | -0.08 [-0.06, -0.03] | 0.01 | <.001 |
| Qualtrics vs. MTurk | 0.03 [-0.01, 0.13] | 0.04 | .08 |  | 0.04 [0.01, 0.15] | 0.04 | .03 |  | 0.04 [0.01, 0.15] | 0.04 | .03 |
| Compassion | 0.12 [0.09, 0.15] | 0.01 | <.001 |  | 0.11 [0.09, 0.14] | 0.01 | <.001 |  | 0.12 [0.09, 0.14] | 0.01 | <.001 |
| Political ideology |  |  |  |  | 0.05 [0.03, 0.08] | 0.01 | <.001 |  | 0.05 [0.03, 0.08] | 0.01 | <.001 |
| Compassion X Political ideology |  |  |  |  |  |  |  |  | 0.01 [-0.01, 0.04] | 0.01 | .35 |
| R^2^ (R^2^ adjusted) | .08(.08) |  |  |  | .08(.08) |  |  |  | .08(.08) |  |  |
| Comparison | △R^2^ = .01, *F_(1,5514)_* = 15.34, *p* < .001 | | | | | |  |  |  |  |  |
|  |  |  |  |  | △R^2^ = .01, *F_(1,5513)_* = 0.88, *p* = .35 | | | | | |  |

*Note.* Age, social desirability, compassion, political ideology scores, and time spent staying home were standardized. *N* = 5533.

**S6 Table.** Predicting Time Spent Home with Multiple Regression in MTurk Sample

|  | Model 1 | | |  | Model 2 | | |  | Model 3 | | |
| --- | --- | --- | --- | --- | --- | --- | --- | --- | --- | --- | --- |
| Predictor | *Β* [95% CI] | *SE* | *p* |  | *Β* [95% CI] | *SE* | *p* |  | *Β* [95% CI] | *SE* | *p* |
| Age | -0.03 [-0.06, 0.01] | .02 | .10 |  | -0.02 [-0.05, 0.01] | .02 | .24 |  | -0.02 [-0.05, 0.01] | .02 | .23 |
| Female | 0.04 [0.01, 0.15] | .03 | .02 |  | 0.04 [0.01, 0.14] | .03 | .02 |  | 0.04 [0.01, 0.14] | .03 | .02 |
| *Race* |  |  |  |  |  |  |  |  |  |  |  |
| Asian | 0.07 [0.13, 0.38] | .06 | <.001 |  | 0.07 [0.13, 0.37] | .06 | <.001 |  | 0.07 [0.13, 0.38] | .06 | <.001 |
| Black | 0.06 [0.10, 0.35] | .06 | <.001 |  | 0.06 [0.09, 0.34] | .06 | .001 |  | 0.06 [0.09, 0.34] | .06 | .001 |
| Other Race | 0.03 [-0.03, 0.26] | .08 | .13 |  | 0.02 [-0.04, 0.26] | .08 | .15 |  | 0.03 [-0.04, 0.26] | .08 | .15 |
| Hispanic/Latino | 0.06 [0.09, 0.34] | .06 | .001 |  | 0.06 [0.09, 0.33] | .06 | .001 |  | 0.06 [0.09, 0.33] | .06 | .001 |
| *Education* |  |  |  |  |  |  |  |  |  |  |  |
| Less than college | 0.01 [-0.06, 0.09] | .04 | .78 |  | 0.01 [-0.06, 0.09] | .04 | .63 |  | 0.01 [-0.06, 0.09] | .04 | .65 |
| More than college | 0.02 [-0.03, 0.15] | .05 | .19 |  | 0.02 [-0.04, 0.14] | .05 | .25 |  | 0.02 [-0.04, 0.14] | .05 | .27 |
| *Employment* |  |  |  |  |  |  |  |  |  |  |  |
| Remote | 0.17 [0.26, 0.42] | .04 | <.001 |  | 0.17 [0.25, 0.41] | .04 | <.001 |  | 0.17 [0.25, 0.41] | .04 | <.001 |
| Unemployed | 0.02 [-0.03, 0.14] | .05 | .23 |  | 0.02 [-0.04, 0.13] | .05 | .33 |  | 0.02 [-0.04, 0.13] | .05 | .31 |
| Income | 0.04 [0.01, 0.09] | .02 | .01 |  | 0.05 [0.01, 0.09] | .02 | .007 |  | 0.05 [0.01, 0.09] | .02 | .007 |
| Social desirability | 0.05 [0.02, 0.09] | .02 | .001 |  | 0.06 [0.02, 0.09] | .02 | .001 |  | 0.06 [0.02, 0.09] | .02 | .001 |
| COVID-19 diagnosis | -0.04 [-0.63, -0.08] | .14 | .01 |  | -0.04 [-0.59, -0.04] | .14 | .03 |  | -0.04 [-0.59, -0.04] | .14 | .03 |
| Increased childcare | 0.06 [0.06, 0.22] | .04 | <.001 |  | 0.06 [0.07, 0.23] | .04 | <.001 |  | 0.06 [0.07, 0.23] | .04 | <.001 |
| Cohort | -0.08 [-0.06, -0.02] | .01 | <.001 |  | -0.08 [-0.06, -0.02] | .01 | <.001 |  | -0.08 [-0.06, -0.02] | .01 | <.001 |
| Compassion | 0.12 [0.09, 0.16] | .02 | <.001 |  | 0.12 [0.08, 0.15] | .02 | <.001 |  | 0.12 [0.09, 0.15] | .02 | <.001 |
| Political ideology |  |  |  |  | 0.05 [0.02, 0.08] | .02 | .003 |  | 0.05 [0.02, 0.08] | .02 | .004 |
| Compassion X Political ideology |  |  |  |  |  |  |  |  | 0.03 [-0.01, 0.06] | .02 | .11 |
| R^2^ (R^2^ adjusted) | .09(.08) |  |  |  | .09(.08) |  |  |  | .09(.08) |  |  |
| Comparison | △R^2^ = .01, *F_(1,3479)_* = 8.65, *p* = .003 | | | | | |  |  |  |  |  |
|  |  |  |  |  | △R^2^ = .01, *F_(1,3478)_* = 2.59, *p* = .11 | | | | | |  |

*Note.* Age, social desirability, compassion, political ideology scores, and time spent staying home were standardized. *N* = 3497.

**S7 Table.** Predicting Time Spent Home with Multiple Regression in Qualtrics Sample

|  | Model 1 | | |  | Model 2 | | |  | Model 3 | | |
| --- | --- | --- | --- | --- | --- | --- | --- | --- | --- | --- | --- |
| Predictor | *Β* [95% CI] | *SE* | *p* |  | *Β* [95% CI] | *SE* | *p* |  | *Β* [95% CI] | *SE* | *p* |
| Age | 0.05 [0.01, 0.11] | .03 | .047 |  | 0.06 [0.01, 0.11] | .03 | .03 |  | 0.06 [0.01, 0.11] | .03 | .03 |
| Female | 0.06 [0.03, 0.21] | .046 | .008 |  | 0.06 [0.03, 0.21] | .05 | .01 |  | 0.06 [0.03, 0.20] | .05 | .01 |
| *Race* |  |  |  |  |  |  |  |  |  |  |  |
| Asian | 0.06 [0.06, 0.51] | .11 | .01 |  | 0.05 [0.05, 0.5] | .11 | .02 |  | 0.05 [0.05, 0.50] | .11 | .02 |
| Black | 0.09 [0.12, 0.41] | .07 | <.001 |  | 0.08 [0.10, 0.39] | .07 | .001 |  | 0.08 [0.10, 0.39] | .07 | .001 |
| Other Race | 0.01 [-0.19, 0.27] | .12 | .73 |  | 0.01 [-0.2, 0.25] | .12 | .83 |  | 0.01 [-0.20, 0.25] | .12 | .83 |
| Hispanic/Latino | 0.13 [0.25, 0.54] | .07 | <.001 |  | 0.14 [0.25, 0.54] | .07 | <.001 |  | 0.14 [0.25, 0.54] | .07 | <.001 |
| *Education* |  |  |  |  |  |  |  |  |  |  |  |
| Less than college | 0.03 [-0.05, 0.17] | .06 | 0.29 |  | 0.03 [-0.04, 0.17] | .06 | .25 |  | 0.03 [-0.04, 0.17] | .06 | .25 |
| More than college | -0.01 [-0.12, 0.10] | .05 | 0.87 |  | -0.01 [-0.12, 0.09] | .05 | .81 |  | -0.01 [-0.12, 0.09] | .05 | 0.81 |
| *Employment* |  |  |  |  |  |  |  |  |  |  |  |
| Remote | 0.16 [0.25, 0.49] | .06 | <.001 |  | 0.16 [0.25, 0.49] | .06 | <.001 |  | 0.16 [0.25, 0.49] | .06 | <.001 |
| Unemployed | 0.01 [-0.09, 0.13] | .06 | .68 |  | 0.01 [-0.1, 0.12] | .06 | .87 |  | 0.01 [-0.10, 0.12] | .06 | .85 |
| Income | 0.02 [-0.02, 0.07] | .02 | .34 |  | 0.02 [-0.03, 0.07] | .02 | .37 |  | 0.02 [-0.03, 0.07] | .02 | .38 |
| Social desirability | 0.04 [-0.01, 0.08] | .02 | .11 |  | 0.04 [-0.01, 0.08] | .02 | .07 |  | 0.04 [-0.01, 0.08] | .02 | .07 |
| COVID-19 diagnosis | -0.06 [-0.79, -0.13] | .17 | .006 |  | -0.06 [-0.77, -0.12] | .17 | .008 |  | -0.06 [-0.77, -0.12] | .17 | .008 |
| Increased childcare | 0.05 [0.02, 0.34] | .08 | .03 |  | 0.05 [0.03, 0.35] | .08 | .02 |  | 0.05 [0.03, 0.35] | .08 | .02 |
| Cohort | -0.08 [-0.12, -0.03] | .02 | .001 |  | -0.09 [-0.12, -0.03] | .02 | .001 |  | -0.09 [-0.12, -0.03] | .02 | .001 |
| Compassion | 0.11 [0.07, 0.16] | .02 | <.001 |  | 0.11 [0.07, 0.15] | .02 | <.001 |  | 0.11 [0.07, 0.15] | .02 | <.001 |
| Political ideology |  |  |  |  | 0.06 [0.02, 0.10] | .02 | .008 |  | 0.06 [0.02, 0.10] | .02 | .007 |
| Compassion X Political ideology |  |  |  |  |  |  |  |  | -0.01 [-0.05, 0.03] | .02 | .57 |
| R^2^ (R^2^ adjusted) | .08(.07) |  |  |  | .08(.07) |  |  |  | .08(.07) |  |  |
| Comparison | △R^2^ = .01, *F_(1,2018)_* = 7.10, *p* = .008 | | | | | |  |  |  |  |  |
|  |  |  |  |  | △R^2^ = .01, *F_(1,2017)_* = 0.33, *p* = .57 | | | | | |  |

*Note.* Age, social desirability, compassion, political ideology scores, and time spent staying home were standardized. *N* = 2036.

**S8 Table.** Predicting Time Spent Keeping a Distance with Multiple Regression

|  | Model 1 | | |  | Model 2 | | |  | Model 3 | | |
| --- | --- | --- | --- | --- | --- | --- | --- | --- | --- | --- | --- |
| Predictor | *Β* [95% CI] | *SE* | *p* |  | *Β* [95% CI] | *SE* | *p* |  | *Β* [95% CI] | *SE* | *p* |
| Age | 0.02 [-0.01, 0.06] | 0.02 | .20 |  | 0.03 [-0.01, 0.07] | 0.02 | .06 |  | 0.03 [-0.01, 0.07] | 0.02 | .06 |
| Female | 0.02 [-0.01, 0.10] | 0.03 | .08 |  | 0.02 [-0.01, 0.10] | 0.03 | .09 |  | 0.02 [-0.01, 0.10] | 0.03 | .09 |
| *Race* |  |  |  |  |  |  |  |  |  |  |  |
| Asian | 0.03 [0.03, 0.25] | 0.06 | .01 |  | 0.03 [0.02, 0.24] | 0.06 | .02 |  | 0.03 [0.02, 0.24] | 0.06 | .02 |
| Black | 0.04 [0.05, 0.23] | 0.05 | .002 |  | 0.04 [0.03, 0.21] | 0.05 | .01 |  | 0.04 [0.03, 0.21] | 0.05 | .009 |
| Other Race | 0.01 [-0.07, 0.18] | 0.06 | .40 |  | 0.01 [-0.08, 0.17] | 0.06 | .52 |  | 0.01 [-0.08, 0.17] | 0.06 | .51 |
| Hispanic/Latino | 0.06 [0.10, 0.28] | 0.05 | <.001 |  | 0.06 [0.10, 0.28] | 0.05 | <.001 |  | 0.06 [0.10, 0.28] | 0.05 | <.001 |
| *Education* |  |  |  |  |  |  |  |  |  |  |  |
| Less than college | 0.03 [0.01, 0.13] | 0.03 | <.001 |  | 0.04 [0.01, 0.14] | 0.03 | .02 |  | 0.04 [0.01, 0.14] | 0.03 | .02 |
| More than college | 0.02 [-0.01, 0.12] | 0.03 | .114 |  | 0.02 [-0.02, 0.12] | 0.03 | .16 |  | 0.02 [-0.02, 0.12] | 0.03 | .17 |
| *Employment* |  |  |  |  |  |  |  |  |  |  |  |
| Remote | 0.09 [0.12, 0.25] | 0.03 | <.001 |  | 0.09 [0.11, 0.25] | 0.03 | <.001 |  | 0.09 [0.11, 0.25] | 0.03 | <.001 |
| Unemployed | -0.02 [-0.12, 0.02] | 0.04 | .18 |  | -0.03 [-0.13, 0.01] | 0.04 | .08 |  | -0.03 [-0.13, 0.01] | 0.04 | .08 |
| Income | 0.04 [0.01, 0.07] | 0.02 | .008 |  | 0.04 [0.01, 0.07] | 0.02 | .005 |  | 0.04 [0.01, 0.07] | 0.02 | .005 |
| Social desirability | 0.04 [0.01, 0.06] | 0.01 | .01 |  | 0.04 [0.01, 0.07] | 0.01 | .004 |  | 0.04 [0.01, 0.07] | 0.01 | .004 |
| COVID-19 diagnosis | -0.05 [-0.64, -0.22] | 0.11 | <.001 |  | -0.05 [-0.6, -0.17] | 0.11 | <.001 |  | -0.05 [-0.60, -0.17] | 0.11 | <.001 |
| Increased childcare | 0.04 [0.04, 0.18] | 0.04 | .003 |  | 0.05 [0.05, 0.19] | 0.04 | .001 |  | 0.05 [0.05, 0.19] | 0.04 | .001 |
| Cohort | -0.07 [-0.05, -0.02] | 0.01 | <.001 |  | -0.07 [-0.05, -0.02] | 0.01 | <.001 |  | -0.07 [-0.05, -0.02] | 0.01 | <.001 |
| Qualtrics vs. MTurk | 0.02 [-0.04, 0.10] | 0.04 | .39 |  | 0.02 [-0.02, 0.12] | 0.04 | .19 |  | 0.02 [-0.02, 0.12] | 0.04 | .19 |
| Compassion | 0.14 [0.12, 0.17] | 0.01 | <.001 |  | 0.14 [0.11, 0.16] | 0.01 | <.001 |  | 0.14 [0.11, 0.16] | 0.01 | <.001 |
| Political ideology |  |  |  |  | 0.07 [0.04, 0.09] | 0.01 | <.001 |  | 0.07 [0.04, 0.09] | 0.01 | <.001 |
| Compassion X Political ideology |  |  |  |  |  |  |  |  | 0.01 [-0.02, 0.03] | 0.01 | .76 |
| R^2^ (R^2^ adjusted) | .05(.05) |  |  |  | .06(.06) |  |  |  | .06(.06) |  |  |
| Comparison | △R^2^ = .01, *F_(1,5514)_* = 23.90, *p* < .001 | | | | | |  |  |  |  |  |
|  |  |  |  |  | △R^2^ = .01, *F_(1,5513)_* = 0.10, *p* = .76 | | | | | |  |

*Note*. Age, social desirability, compassion, and political ideology scores, and time spent keeping a distance were standardized. N = 5533.

**S9 Table.** Predicting Frequency of Mask Wearing with Multiple Regression

|  | Model 1 | | |  | Model 2 | | |  | Model 3 | | |
| --- | --- | --- | --- | --- | --- | --- | --- | --- | --- | --- | --- |
| Predictor | *Β* [95% CI] | *SE* | *p* |  | *Β* [95% CI] | *SE* | *p* |  | *Β* [95% CI] | *SE* | *p* |
| Age | 0.14 [0.08, 0.19] | 0.03 | <.001 |  | 0.18 [0.12, 0.23] | 0.03 | <.001 |  | 0.18 [0.12, 0.24] | 0.03 | <.001 |
| Female | 0.05 [0.01, 0.19] | 0.05 | .04 |  | 0.04 [-0.01, 0.16] | 0.05 | .10 |  | 0.04 [-0.02, 0.16] | 0.05 | .10 |
| *Race* |  |  |  |  |  |  |  |  |  |  |  |
| Asian | 0.07 [0.11, 0.56] | 0.12 | .004 |  | 0.06 [0.08, 0.52] | 0.11 | .007 |  | 0.06 [0.08, 0.52] | 0.11 | .007 |
| Black | 0.07 [0.06, 0.33] | 0.07 | .003 |  | 0.05 [0.01, 0.26] | 0.07 | .05 |  | 0.05 [-0.01, 0.25] | 0.07 | .06 |
| Other Race | 0.03 [-0.07, 0.35] | 0.11 | .19 |  | 0.03 [-0.06, 0.35] | 0.10 | .16 |  | 0.03 [-0.06, 0.35] | 0.10 | .17 |
| Hispanic/Latino | 0.08 [0.08, 0.37] | 0.07 | .002 |  | 0.08 [0.10, 0.38] | 0.07 | .001 |  | 0.08 [0.10, 0.38] | 0.07 | .001 |
| *Education* |  |  |  |  |  |  |  |  |  |  |  |
| Less than college | -0.10 [-0.31, -0.10] | 0.06 | <.001 |  | -0.08 [-0.28, -0.07] | 0.05 | .001 |  | -0.08 [-0.28, -0.07] | 0.05 | .001 |
| More than college | 0.01 [-0.09, 0.14] | 0.06 | .62 |  | 0.01 [-0.11, 0.12] | 0.06 | .95 |  | 0.01 [-0.11, 0.12] | 0.06 | .94 |
| *Employment* |  |  |  |  |  |  |  |  |  |  |  |
| Remote | 0.09 [0.08, 0.31] | 0.06 | .001 |  | 0.08 [0.06, 0.28] | 0.06 | .003 |  | 0.08 [0.06, 0.28] | 0.06 | .003 |
| Unemployed | 0.05 [-0.01, 0.23] | 0.06 | .06 |  | 0.02 [-0.07, 0.16] | 0.06 | .49 |  | 0.02 [-0.07, 0.16] | 0.06 | .48 |
| Income | -0.01 [-0.06, 0.04] | 0.03 | .66 |  | -0.01 [-0.05, 0.05] | 0.03 | .87 |  | -0.01 [-0.05, 0.05] | 0.03 | .88 |
| Social desirability | 0.04 [-0.01, 0.09] | 0.02 | .08 |  | 0.06 [0.02, 0.11] | 0.02 | .01 |  | 0.06 [0.02, 0.11] | 0.02 | .009 |
| COVID-19 diagnosis | 0.01 [-0.31, 0.33] | 0.16 | .95 |  | 0.01 [-0.25, 0.38] | 0.16 | .67 |  | 0.01 [-0.25, 0.37] | 0.16 | .71 |
| Increased childcare | -0.04 [-0.23, 0.02] | 0.06 | .10 |  | -0.01 [-0.16, 0.08] | 0.06 | .54 |  | -0.01 [-0.16, 0.09] | 0.06 | .55 |
| Cohort | 0.05 [0.01, 0.20] | 0.05 | .034 |  | 0.04 [-0.02, 0.17] | 0.05 | .10 |  | 0.04 [-0.01, 0.17] | 0.05 | .10 |
| Qualtrics vs. MTurk | 0.04 [-0.04, 0.19] | 0.06 | .204 |  | 0.06 [0.01, 0.22] | 0.06 | .048 |  | 0.05 [-0.01, 0.22] | 0.06 | .05 |
| Compassion | 0.13 [0.09, 0.18] | 0.02 | <.001 |  | 0.11 [0.06, 0.15] | 0.02 | <.001 |  | 0.10 [0.06, 0.15] | 0.02 | <.001 |
| Political ideology |  |  |  |  | 0.23 [0.19, 0.28] | 0.02 | <.001 |  | 0.23 [0.19, 0.28] | 0.02 | <.001 |
| Compassion X Political ideology |  |  |  |  |  |  |  |  | -0.02 [-0.06, 0.02] | 0.02 | .32 |
| R^2^ (R^2^ adjusted) | .10(.09) |  |  |  | .14 (.14) |  |  |  | .14 (.14) |  |  |
| Comparison | △R^2^ = .05, *F_(1,1848)_* = 102.18, *p* < .001 | | | | | |  |  |  |  |  |
|  |  |  |  |  | △R^2^ = .01, *F_(1,1847)_* = 0.99, *p* = .32 | | | | | |  |

*Note*. Age, social desirability, compassion, and political ideology scores, and mask wearing were standardized. N = 1867.

**S10 Table.** Predicting Understanding of Others’ Fear with Multiple Regression

|  | Model 1 | | |  | Model 2 | | |  | Model 3 | | |
| --- | --- | --- | --- | --- | --- | --- | --- | --- | --- | --- | --- |
| Predictor | *Β* [95% CI] | *SE* | *p* |  | *Β* [95% CI] | *SE* | *p* |  | *Β* [95% CI] | *SE* | *p* |
| Age | 0.02 [-0.01, 0.05] | 0.02 | .20 |  | 0.05 [0.02, 0.09] | 0.02 | .001 |  | 0.05 [0.02, 0.09] | 0.02 | .001 |
| Female | -0.01 [-0.07, 0.03] | 0.03 | .46 |  | -0.01 [-0.07, 0.04] | 0.03 | .54 |  | -0.01 [-0.07, 0.03] | 0.03 | .52 |
| *Race* |  |  |  |  |  |  |  |  |  |  |  |
| Asian | 0.04 [0.08, 0.29] | 0.05 | .001 |  | 0.04 [0.04, 0.25] | 0.05 | .006 |  | 0.04 [0.04, 0.25] | 0.05 | .007 |
| Black | 0.02 [-0.03, 0.15] | 0.05 | .17 |  | 0.01 [-0.09, 0.09] | 0.04 | .96 |  | 0.01 [-0.08, 0.09] | 0.05 | .94 |
| Other Race | 0.01 [-0.09, 0.16] | 0.06 | .57 |  | -0.01 [-0.13, 0.11] | 0.06 | .92 |  | -0.01 [-0.13, 0.11] | 0.06 | .86 |
| Hispanic/Latino | 0.01 [-0.09, 0.09] | 0.05 | .94 |  | -0.02 [-0.09, 0.08] | 0.04 | .88 |  | -0.01 [-0.09, 0.08] | 0.05 | .88 |
| *Education* |  |  |  |  |  |  |  |  |  |  |  |
| Less than college | -0.03 [-0.12, 0.01] | 0.03 | .08 |  | -0.01 [-0.09, 0.03] | 0.03 | .37 |  | -0.01 [-0.09, 0.03] | 0.03 | .33 |
| More than college | 0.03 [-0.01, 0.13] | 0.03 | .07 |  | 0.02 [-0.03, 0.11] | 0.03 | .23 |  | 0.02 [-0.02, 0.11] | 0.03 | .21 |
| *Employment* |  |  |  |  |  |  |  |  |  |  |  |
| Remote | 0.07 [0.08, 0.21] | 0.03 | <.001 |  | 0.06 [0.06, 0.19] | 0.03 | <.001 |  | 0.06 [0.07, 0.20] | 0.03 | <.001 |
| Unemployed | 0.10 [0.15, 0.28] | 0.03 | <.001 |  | 0.08 [0.11, 0.24] | 0.03 | <.001 |  | 0.08 [0.11, 0.24] | 0.03 | <.001 |
| Income | -0.01 [-0.03, 0.03] | 0.01 | .81 |  | 0.01 [-0.02, 0.03] | 0.01 | .70 |  | 0.01 [-0.02, 0.03] | 0.01 | .76 |
| Social desirability | -0.08 [-0.11, -0.06] | 0.01 | <.001 |  | -0.07 [-0.09, -0.04] | 0.01 | <.001 |  | -0.07 [-0.09, -0.04] | 0.01 | <.001 |
| COVID-19 diagnosis | -0.03 [-0.48, -0.07] | 0.11 | .01 |  | -0.02 [-0.39, 0.02] | 0.10 | .08 |  | -0.02 [-0.36, 0.05] | 0.11 | .14 |
| Increased childcare | -0.03 [-0.15, -0.01] | 0.04 | .03 |  | -0.01 [-0.10, 0.03] | 0.03 | .30 |  | -0.01 [-0.1, 0.03] | 0.04 | .32 |
| Cohort | -0.03 [-0.03, -.01] | 0.01 | .02 |  | -0.04 [-0.03, -0.01] | 0.01 | .01 |  | -0.04 [-0.03, -0.01] | 0.01 | .01 |
| Qualtrics vs. MTurk | -0.08 [-0.23, -0.09] | 0.04 | <.001 |  | -0.06 [-0.19, -0.05] | 0.04 | .001 |  | -0.06 [-0.19, -0.05] | 0.04 | .001 |
| Empathy | 0.26 [0.23, 0.29] | 0.01 | <.001 |  | 0.23 [0.20, 0.26] | 0.01 | <.001 |  | 0.23 [0.20, 0.25] | 0.01 | <.001 |
| Political ideology |  |  |  |  | 0.20 [0.17, 0.23] | 0.01 | <.001 |  | 0.20 [0.18, 0.23] | 0.01 | <.001 |
| Empathy X Political ideology |  |  |  |  |  |  |  |  | -0.05 [-0.07, -0.02] | 0.01 | <.001 |
| R^2^ (R^2^ adjusted) | .10(.10) |  |  |  | .13(.13) |  |  |  | .14(.13) |  |  |
| Comparison | △R^2^ = .10, *F_(1,5514)_* = 224.66, *p* < .001 | | | | | |  |  |  |  |  |
|  |  |  |  |  | △R^2^ = .01, *F_(1,5513)_* = 13.17, *p* < .001 | | | | | |  |

*Note*. Age, social desirability, compassion, and political ideology scores, and understanding were standardized. N = 5533.

**S11 Table.** Predicting Understanding of Others’ Fear with Multiple Regression for MTurk Participants

|  | Model 1 | | |  | Model 2 | | |  | Model 3 | | |
| --- | --- | --- | --- | --- | --- | --- | --- | --- | --- | --- | --- |
| Predictor | *Β* [95% CI] | *SE* | *p* |  | *Β* [95% CI] | *SE* | *p* |  | *Β* [95% CI] | *SE* | *p* |
| Age | -0.01 [-0.04, 0.03] | .02 | .63 |  | 0.03 [-0.01, 0.06] | .03 | .14 |  | 0.03 [-0.01, 0.06] | .02 | .13 |
| Female | -0.03 [-0.14, -0.01] | .03 | .04 |  | -0.03 [-0.13, -0.01] | -.03 | .04 |  | -0.03 [-0.13, -0.01] | .03 | .04 |
| *Race* |  |  |  |  |  |  |  |  |  |  |  |
| Asian | 0.04 [0.03, 0.28] | .06 | .01 |  | 0.03 [-0.01, 0.24] | .03 | .05 |  | 0.03 [-0.01, 0.24] | .06 | .06 |
| Black | 0.01 [-0.08, 0.16] | .06 | .55 |  | -0.01 [-0.13, 0.11] | -.01 | .84 |  | -0.01 [-0.13, 0.11] | .06 | .85 |
| Other Race | 0.01 [-0.11, 0.19] | .08 | .60 |  | 0.01 [-0.14, 0.15] | .01 | .93 |  | 0.01 [-0.15, 0.14] | .07 | .99 |
| Hispanic/Latino | 0.01 [-0.11, 0.13] | .06 | .90 |  | -0.01 [-0.13, 0.10] | -.01 | .81 |  | -0.01 [-0.13, 0.11] | .06 | .83 |
| *Education* |  |  |  |  |  |  |  |  |  |  |  |
| Less than college | -0.01 [-0.10, 0.05] | .04 | .56 |  | 0.01 [-0.06, 0.09] | .01 | .73 |  | 0.01 [-0.06, 0.08] | .04 | .78 |
| More than college | 0.03 [-0.02, 0.15] | .04 | .14 |  | 0.02 [-0.05, 0.12] | .02 | .38 |  | 0.02 [-0.04, 0.12] | .04 | .35 |
| *Employment* |  |  |  |  |  |  |  |  |  |  |  |
| Remote | 0.07 [0.06, 0.22] | .04 | <.001 |  | 0.06 [0.05, 0.21] | .07 | .001 |  | 0.07 [0.05, 0.21] | .04 | .001 |
| Unemployed | 0.08 [0.11, 0.28] | .05 | <.001 |  | 0.07 [0.07, 0.25] | .07 | <.001 |  | 0.07 [0.07, 0.24] | .04 | <.001 |
| Income | -0.01 [-0.05, 0.03] | .02 | .52 |  | 0.01 [-0.03, 0.04] | .01 | .72 |  | 0.01 [-0.03, 0.04] | .02 | .77 |
| Social desirability | -0.08 [-0.11, -0.05] | .02 | <.001 |  | -0.06 [-0.10, -0.03] | -.06 | <.001 |  | -0.06 [-0.10, -0.03] | .02 | <.001 |
| COVID-19 diagnosis | -0.05 [-0.71, -0.16] | .14 | .002 |  | -0.03 [-0.56, -0.02] | -.03 | .04 |  | -0.03 [-0.51, 0.03] | .14 | .08 |
| Increased childcare | -0.03 [-0.15, 0.01] | .04 | .08 |  | -0.01 [-0.10, 0.05] | -.01 | .51 |  | -0.01 [-0.10, 0.05] | .04 | .56 |
| Cohort | -0.04 [-0.04, -0.01] | .008 | .02 |  | -0.04 [-0.03, -0.01] | -.04 | .02 |  | -0.04 [-0.03, -0.01] | .01 | .02 |
| Empathy | 0.30 [0.26, 0.33] | .02 | <.001 |  | 0.26 [0.23, 0.30] | .26 | <.001 |  | 0.26 [0.23, 0.29] | .02 | <.001 |
| Political ideology |  |  |  |  | 0.21 [0.17, 0.24] | .21 | <.001 |  | 0.21 [0.18, 0.24] | .02 | <.001 |
| Empathy X Political ideology |  |  |  |  |  |  |  |  | -0.05 [-0.08, -0.02] | .02 | .001 |
| R^2^ (R^2^ adjusted) | .10(.10) |  |  |  | .14(.14) |  |  |  | .14(.14) |  |  |
| Comparison | △R^2^ = .04, *F_(1,3479)_* = 155.57, *p* < .001 | | | | | |  |  |  |  |  |
|  |  |  |  |  | △R^2^ = .01, *F_(1,3478)_* = 10.67, *p* = .001 | | | | | |  |

*Note*. Age, social desirability, compassion, and political ideology scores, and understanding were standardized. N = 3497.

**S12 Table.** Predicting Understanding of Others’ Fear with Multiple Regression for Qualtrics Participants

|  | Model 1 | | |  | Model 2 | | |  | Model 3 | | |
| --- | --- | --- | --- | --- | --- | --- | --- | --- | --- | --- | --- |
| Predictor | *Β* [95% CI] | *SE* | *p* |  | *Β* [95% CI] | *SE* | *p* |  | *Β* [95% CI] | *SE* | *p* |
| Age | 0.07 [0.02, 0.13] | .03 | .01 |  | 0.09 [0.04, 0.14] | .03 | .001 |  | 0.09 [0.04, 0.15] | .03 | .001 |
| Female | 0.04 [-0.01, 0.17] | .05 | .08 |  | 0.04 [-0.01, 0.17] | .05 | .09 |  | 0.04 [-0.01, 0.17] | .05 | .10 |
| *Race* |  |  |  |  |  |  |  |  |  |  |  |
| Asian | 0.06 [0.06, 0.51] | .11 | .01 |  | 0.05 [0.02, 0.46] | .11 | .03 |  | 0.05 [0.02, 0.46] | .11 | .03 |
| Black | 0.03 [-0.06, 0.23] | .07 | .23 |  | 0.01 [-0.11, 0.17] | .07 | .68 |  | 0.01 [-0.11, 0.17] | .07 | .65 |
| Other Race | 0.01 [-0.21, 0.24] | .12 | .92 |  | -0.01 [-0.27, 0.18] | .11 | .69 |  | -0.01 [-0.27, 0.18] | .11 | .68 |
| Hispanic/Latino | 0.01 [-0.11, 0.17] | .07 | .69 |  | 0.01 [-0.100, 0.18] | .07 | .61 |  | 0.01 [-0.11, 0.18] | .07 | .61 |
| *Education* |  |  |  |  |  |  |  |  |  |  |  |
| Less than college | -0.05 [-0.22, -0.01] | .06 | .04 |  | -0.05 [-0.2, 0.01] | .06 | .08 |  | -0.05 [-0.21, 0.01] | .06 | .07 |
| More than college | 0.02 [-0.06, 0.16] | .05 | .36 |  | 0.02 [-0.07, 0.14] | .05 | .53 |  | 0.02 [-0.07, 0.14] | .05 | .53 |
| *Employment* |  |  |  |  |  |  |  |  |  |  |  |
| Remote | 0.07 [0.04, 0.28] | .06 | .009 |  | 0.06 [0.03, 0.26] | .06 | .03 |  | 0.07 [0.03, 0.27] | .06 | .02 |
| Unemployed | 0.11 [0.10, 0.32] | .06 | <.001 |  | 0.08 [0.06, 0.28] | .06 | .002 |  | 0.09 [0.06, 0.28] | .06 | .002 |
| Income | 0.01 [-0.04, 0.05] | .02 | .90 |  | -0.01 [-0.05, 0.05] | .02 | .97 |  | -0.01 [-0.05, 0.04] | .02 | .94 |
| Social desirability | -0.08 [-0.13, -0.04] | .02 | <.001 |  | -0.07 [-0.11, -0.02] | .02 | .002 |  | -0.07 [-0.11, -0.02] | .02 | .002 |
| COVID-19 diagnosis | -0.01 [-0.39, 0.26] | .17 | .70 |  | -0.01 [-0.35, 0.29] | .17 | .85 |  | -0.01 [-0.35, 0.30] | .17 | .87 |
| Increased childcare | -0.02 [-0.24, 0.08] | 0.08 | .33 |  | -0.02 [-0.21, 0.10] | .08 | .49 |  | -0.02 [-0.21, 0.10] | .08 | .48 |
| Cohort | -0.02 [-0.06, 0.02] | .02 | .39 |  | -0.03 [-0.07, 0.01] | .02 | .17 |  | -0.03 [-0.07, 0.01] | .02 | .18 |
| Empathy | 0.22 [0.17, 0.26] | .02 | <.001 |  | 0.19 [0.14, 0.23] | .02 | <.001 |  | 0.19 [0.14, 0.23] | .02 | <.001 |
| Political ideology |  |  |  |  | 0.19 [0.14, 0.23] | .02 | <.001 |  | 0.19 [0.15, 0.23] | .02 | <.001 |
| Empathy X Political ideology |  |  |  |  |  |  |  |  | -0.03 [-0.07, 0.01] | .02 | .17 |
| R^2^ (R^2^ adjusted) | .08(.07) |  |  |  | .11(.10) |  |  |  | .11(.10) |  |  |
| Comparison | △R^2^ = .03, *F_(1,2018)_* = 73.10, *p* < .001 | | | | | |  |  |  |  |  |
|  |  |  |  |  | △R^2^ = .01, *F_(1,2017)_* = 1.93, *p* = .17 | | | | | |  |

*Note.* Age, social desirability, compassion, political ideology scores, and time understanding were standardized. *N* = 2036.

**S13 Table.** Predicting View of Others Overreacting with Multiple Regression

|  | Model 1 | | |  | Model 2 | | |  | Model 3 | | |
| --- | --- | --- | --- | --- | --- | --- | --- | --- | --- | --- | --- |
| Predictor | *Β* [95% CI] | *SE* | *p* |  | *Β* [95% CI] | *SE* | *p* |  | *Β* [95% CI] | *SE* | *p* |
| Age | -0.02 [-0.05, 0.01] | 0.02 | .21 |  | -0.08 [-0.11, -0.05] | 0.02 | <.001 |  | -0.08 [-0.11, -0.05] | 0.02 | <.001 |
| Female | -0.04 [-0.13, -0.03] | 0.03 | .004 |  | -0.04 [-0.13, -0.04] | 0.02 | .001 |  | -0.04 [-0.13, -0.04] | 0.02 | .001 |
| *Race* |  |  |  |  |  |  |  |  |  |  |  |
| Asian | -0.06 [-0.33, -0.12] | 0.05 | <.001 |  | -0.04 [-0.26, -0.06] | 0.05 | .002 |  | -0.04 [-0.26, -0.06] | 0.05 | .002 |
| Black | -0.07 [-0.32, -0.15] | 0.05 | <.001 |  | -0.04 [-0.21, -0.04] | 0.04 | .003 |  | -0.04 [-0.21, -0.04] | 0.04 | .003 |
| Other Race | -0.02 [-0.22, 0.03] | 0.06 | .14 |  | -0.01 [-0.13, 0.10] | 0.06 | .80 |  | -0.01 [-0.13, 0.10] | 0.06 | .82 |
| Hispanic/Latino | -0.02 [-0.16, 0.01] | 0.05 | .10 |  | -0.02 [-0.14, 0.03] | 0.04 | .19 |  | -0.02 [-0.14, 0.03] | 0.04 | .19 |
| *Education* |  |  |  |  |  |  |  |  |  |  |  |
| Less than college | 0.04 [0.03, 0.15] | 0.03 | .006 |  | 0.02 [-0.02, 0.09] | 0.03 | .24 |  | 0.02 [-0.02, 0.09] | 0.03 | .23 |
| More than college | -0.01 [-0.07, 0.06] | 0.03 | .83 |  | 0.01 [-0.03, 0.09] | 0.03 | .32 |  | 0.01 [-0.03, 0.09] | 0.03 | .33 |
| *Employment* |  |  |  |  |  |  |  |  |  |  |  |
| Remote | -0.07 [-0.21, -0.08] | 0.03 | <.001 |  | -0.06 [-0.18, -0.05] | 0.03 | <.001 |  | -0.06 [-0.18, -0.06] | 0.03 | <.001 |
| Unemployed | -0.10 [-0.27, -0.14] | 0.03 | <.001 |  | -0.06 [-0.19, -0.07] | 0.03 | <.001 |  | -0.06 [-0.19, -0.07] | 0.03 | <.001 |
| Income | 0.02 [-0.01, 0.05] | 0.01 | .12 |  | 0.01 [-0.02, 0.03] | 0.01 | .67 |  | 0.01 [-0.02, 0.03] | 0.01 | .65 |
| Social desirability | 0.03 [0.01, 0.05] | 0.01 | .04 |  | -0.01 [-0.03, 0.02] | 0.01 | .70 |  | -0.01 [-0.03, 0.02] | 0.01 | .70 |
| COVID-19 diagnosis | 0.08 [0.45, 0.87] | 0.11 | <.001 |  | 0.06 [0.30, 0.68] | 0.10 | <.001 |  | 0.06 [0.29, 0.67] | 0.10 | <.001 |
| Increased childcare | 0.04 [0.04, 0.18] | 0.04 | .002 |  | 0.01 [-0.03, 0.10] | 0.03 | .24 |  | 0.01 [-0.03, 0.10] | 0.03 | .25 |
| Cohort | -0.01 [-0.02, 0.01] | 0.01 | .78 |  | -0.01 [-0.02, 0.01] | 0.01 | .81 |  | -0.01 [-0.02, 0.01] | 0.01 | .80 |
| Empathy | -0.25 [-0.28, -0.23] | 0.01 | <.001 |  | -0.19 [-0.22, -0.17] | 0.01 | <.001 |  | -0.19 [-0.22, -0.17] | 0.01 | <.001 |
| Political ideology |  |  |  |  | -0.37 [-0.39, -0.34] | 0.01 | <.001 |  | -0.37 [-0.39, -0.34] | 0.01 | <.001 |
| Empathy X Political ideology |  |  |  |  |  |  |  |  | 0.02 [-0.01, 0.04] | 0.01 | .19 |
| R^2^ (R^2^ adjusted) | .10(.10) |  |  |  | .22(.22) |  |  |  | .22(.22) |  |  |
| Comparison | △R^2^ = .12, *F_(1,5514)_* = 857.99, *p* < .001 | | | | | |  |  |  |  |  |
|  |  |  |  |  | △R^2^ = .01, *F_(1,5513)_* = 1.72, *p* = .19 | | | | | |  |

*Note*. Age, social desirability, compassion, and political ideology scores, and viewing others as overreacting were standardized. N = 5533.

**S14 Table.** Predicting Belief that Staying Home Prevents Spread with Multiple Regression

|  | Model 1 | | |  | Model 2 | | |  | Model 3 | | |
| --- | --- | --- | --- | --- | --- | --- | --- | --- | --- | --- | --- |
| Predictor | *Β* [95% CI] | *SE* | *p* |  | *Β* [95% CI] | *SE* | *p* |  | *Β* [95% CI] | *SE* | *p* |
| Age | -0.03 [-0.07, 0.01] | 0.02 | .07 |  | 0.01 [-0.03, 0.04] | 0.02 | .94 |  | 0.01 [-0.03, 0.04] | 0.02 | .94 |
| Female | 0.01 [-0.06, 0.06] | 0.03 | .94 |  | 0.01 [-0.05, 0.07] | 0.03 | .77 |  | 0.01 [-0.05, 0.06] | 0.03 | .78 |
| *Race* |  |  |  |  |  |  |  |  |  |  |  |
| Asian | 0.05 [0.08, 0.34] | 0.07 | .001 |  | 0.04 [0.03, 0.29] | 0.06 | .01 |  | 0.04 [0.03, 0.28] | 0.06 | .01 |
| Black | 0.01 [-0.06, 0.13] | 0.05 | .49 |  | -0.01 [-0.13, 0.06] | 0.05 | .42 |  | -0.01 [-0.13, 0.06] | 0.05 | .43 |
| Other Race | 0.02 [-0.03, 0.25] | 0.07 | .14 |  | 0.01 [-0.07, 0.20] | 0.07 | .37 |  | 0.01 [-0.08, 0.20] | 0.07 | .39 |
| Hispanic/Latino | 0.01 [-0.08, 0.13] | 0.05 | .65 |  | 0.01 [-0.09, 0.11] | 0.05 | .80 |  | 0.01 [-0.09, 0.11] | 0.05 | .80 |
| *Education* |  |  |  |  |  |  |  |  |  |  |  |
| Less than college | -0.02 [-0.12, 0.02] | 0.03 | .15 |  | -0.01 [-0.09, 0.05] | 0.03 | .59 |  | -0.01 [-0.09, 0.05] | 0.03 | .57 |
| More than college | 0.01 [-0.04, 0.11] | 0.04 | .38 |  | 0.01 [-0.06, 0.08] | 0.04 | .78 |  | 0.01 [-0.06, 0.08] | 0.04 | .76 |
| *Employment* |  |  |  |  |  |  |  |  |  |  |  |
| Remote | 0.11 [0.16, 0.31] | 0.04 | <.001 |  | 0.11 [0.14, 0.29] | 0.04 | <.001 |  | 0.11 [0.15, 0.29] | 0.04 | <.001 |
| Unemployed | 0.09 [0.12, 0.27] | 0.04 | <.001 |  | 0.07 [0.07, 0.22] | 0.04 | <.001 |  | 0.07 [0.07, 0.22] | 0.04 | <.001 |
| Income | -0.02 [-0.06, 0.01] | 0.02 | .17 |  | -0.01 [-0.05, 0.02] | 0.02 | .35 |  | -0.01 [-0.05, 0.02] | 0.02 | .33 |
| Social desirability | 0.01 [-0.02, 0.04] | 0.02 | .33 |  | 0.04 [0.01, 0.07] | 0.01 | .01 |  | 0.04 [0.01, 0.07] | 0.01 | .01 |
| COVID-19 diagnosis | -0.03 [-0.46, -0.01] | 0.11 | .04 |  | -0.02 [-0.37, 0.07] | 0.11 | .19 |  | -0.02 [-0.35, 0.09] | 0.11 | .24 |
| Increased childcare | 0.01 [-0.08, 0.08] | 0.04 | .96 |  | 0.02 [-0.03, 0.13] | 0.04 | .20 |  | 0.02 [-0.03, 0.13] | 0.04 | .20 |
| Cohort | -0.02 [-0.04, 0.01] | 0.01 | .10 |  | -0.03 [-0.04, 0.01] | 0.01 | .05 |  | -0.03 [-0.04, 0.01] | 0.01 | .06 |
| Qualtrics vs. MTurk | 0.04 [-0.01, 0.15] | 0.04 | .05 |  | 0.06 [0.05, 0.19] | 0.04 | .001 |  | 0.06 [0.05, 0.19] | 0.04 | .001 |
| Empathy | 0.23 [0.20, 0.26] | 0.02 | <.001 |  | 0.20 [0.16, 0.22] | 0.02 | <.001 |  | 0.20 [0.16, 0.22] | 0.02 | <.001 |
| Political ideology |  |  |  |  | 0.23 [0.20, 0.25] | 0.01 | <.001 |  | 0.23 [0.20, 0.25] | 0.01 | <.001 |
| Empathy X Political ideology |  |  |  |  |  |  |  |  | -0.02 [-0.05, 0.01] | 0.01 | .09 |
| R^2^ (R^2^ adjusted) | .07(.07) |  |  |  | .12(.11) |  |  |  | .12(.11) |  |  |
| Comparison | △R^2^ = .05, *F_(1,4616)_* = 235.36, *p* < .001 | | | | | |  |  |  |  |  |
|  |  |  |  |  | △R^2^ = .01, *F_(1,4615)_* = 2.82, *p* = .09 | | | | | |  |

*Note*. Age, social desirability, compassion, and political ideology scores, and efficacy beliefs were standardized. N = 4635.

**S15 Table.** Predicting Belief that Staying Home Prevents Spread with Multiple Regression for MTurk Participants

|  | Model 1 | | |  | Model 2 | | |  | Model 3 | | |
| --- | --- | --- | --- | --- | --- | --- | --- | --- | --- | --- | --- |
| Predictor | *Β* [95% CI] | *SE* | *p* |  | *Β* [95% CI] | *SE* | *p* |  | *Β* [95% CI] | *SE* | *p* |
| Age | -0.04 [-0.08, 0.01] | .02 | .08 |  | 0.01 [-0.03, 0.04] | .02 | .85 |  | 0.01 [-0.03, 0.04] | .02 | .81 |
| Female | 0.03 [-0.03, 0.13] | .04 | .20 |  | 0.02 [-0.03, 0.12] | .04 | .22 |  | 0.02 [-0.03, 0.12] | .04 | .22 |
| *Race* |  |  |  |  |  |  |  |  |  |  |  |
| Asian | 0.05 [0.07, 0.39] | .08 | .006 |  | 0.04 [0.01, 0.32] | .08 | .04 |  | 0.04 [0.01, 0.31] | .08 | .05 |
| Black | -0.02 [-0.22, 0.07] | .07 | .30 |  | -0.04 [-0.29, -0.01] | .07 | .03 |  | -0.04 [-0.29, -0.01] | .07 | .03 |
| Other Race | 0.03 [-0.03, 0.33] | .09 | .11 |  | 0.03 [-0.05, 0.30] | .09 | .17 |  | 0.02 [-0.06, 0.30] | .09 | .19 |
| Hispanic/Latino | -0.01 [-0.21, 0.11] | .08 | .54 |  | -0.02 [-0.23, 0.07] | .08 | .28 |  | -0.02 [-0.23, 0.07] | .08 | .29 |
| *Education* |  |  |  |  |  |  |  |  |  |  |  |
| Less than college | -0.01 [-0.12, 0.06] | .05 | .51 |  | 0.01 [-0.07, 0.10] | .04 | .80 |  | 0.01 [-0.07, 0.10] | .04 | .71 |
| More than college | 0.01 [-0.10, 0.11] | .05 | .92 |  | -0.01 [-0.13, 0.07] | .05 | .54 |  | -0.01 [-0.12, 0.07] | .05 | .63 |
| *Employment* |  |  |  |  |  |  |  |  |  |  |  |
| Remote | 0.12 [0.15, 0.34] | .05 | <.001 |  | 0.11 [0.13, 0.31] | .05 | <.001 |  | 0.11 [0.13, 0.31] | .05 | <.001 |
| Unemployed | 0.11 [0.15, 0.36] | .05 | <.001 |  | 0.09 [0.10, 0.30] | .05 | <.001 |  | 0.08 [0.10, 0.30] | .05 | <.001 |
| Income | 0.01 [-0.03, 0.06] | .02 | .64 |  | 0.03 [-0.01, 0.08] | .02 | .17 |  | 0.03 [-0.01, 0.08] | .02 | .14 |
| Social desirability | 0.03 [-0.01, 0.06] | .02 | .19 |  | 0.05 [0.01, 0.09] | .02 | .008 |  | 0.05 [0.01, 0.09] | .02 | .008 |
| COVID-19 diagnosis | -0.09 [-1.04, -0.41] | .16 | <.001 |  | -0.06 [-0.81, -0.21] | .15 | .001 |  | -0.06 [-0.8, -0.20] | .15 | .001 |
| Increased childcare | -0.01 [-0.12, 0.06] | .05 | .46 |  | 0.01 [-0.06, 0.12] | .05 | .48 |  | 0.01 [-0.05, 0.12] | .04 | .46 |
| Cohort | -0.07 [-0.07, -0.02] | .01 | .001 |  | -0.06 [-0.07, -0.02] | .01 | <.001 |  | -0.06 [-0.07, -0.02] | .01 | .001 |
| Empathy | 0.18 [0.14, 0.22] | .02 | <.001 |  | 0.15 [0.11, 0.19] | .02 | <.001 |  | 0.15 [0.11, 0.18] | .02 | <.001 |
| Political ideology |  |  |  |  | 0.27 [0.23, 0.30] | .02 | <.001 |  | 0.27 [0.23, 0.31] | .02 | <.001 |
| Empathy X Political ideology |  |  |  |  |  |  |  |  | -0.06 [-0.1, -0.03] | .02 | .001 |
| R^2^ (R^2^ adjusted) | .07(.06) |  |  |  | .13(.13) |  |  |  | .14(.13) |  |  |
| Comparison | △R^2^ = .07, *F_(1,2581)_* = 192.35, *p* < .001 | | | | | |  |  |  |  |  |
|  |  |  |  |  | △R^2^ = .01, *F_(1,2580)_* = 11.80, *p* = .01 | | | | | |  |

*Note*. Age, social desirability, compassion, and political ideology scores, and the belief that staying home prevents spread were standardized. N = 2599.

**S16 Table.** Predicting Belief that Staying Home Prevents Spread with Multiple Regression for Qualtrics Participants

|  | Model 1 | | |  | Model 2 | | |  | Model 3 | | |
| --- | --- | --- | --- | --- | --- | --- | --- | --- | --- | --- | --- |
| Predictor | *Β* [95% CI] | *SE* | *p* |  | *Β* [95% CI] | *SE* | *p* |  | *Β* [95% CI] | *SE* | *p* |
| Age | 0.01 [-0.04, 0.07] | .03 | .72 |  | 0.03 [-0.02, 0.08] | .03 | .28 |  | 0.03 [-0.02, 0.08] | .03 | .28 |
| Female | 0.01 [-0.06, 0.12] | .05 | .57 |  | 0.01 [-0.07, 0.11] | .05 | .63 |  | 0.01 [-0.07, 0.11] | .05 | .63 |
| *Race* |  |  |  |  |  |  |  |  |  |  |  |
| Asian | 0.04 [-0.03, 0.42] | .11 | .09 |  | 0.03 [-0.07, 0.37] | .11 | .17 |  | 0.03 [-0.07, 0.38] | .11 | .17 |
| Black | 0.02 [-0.10, 0.19] | .07 | .53 |  | 0 [-0.15, 0.13] | .07 | .88 |  | -0.01 [-0.15, 0.13] | .07 | .88 |
| Other Race | -0.01 [-0.23, 0.22] | .12 | .97 |  | -0.01 [-0.28, 0.16] | .11 | .60 |  | -0.01 [-0.28, 0.16] | .11 | .60 |
| Hispanic/Latino | 0.01 [-0.12, 0.17] | .07 | .72 |  | 0.01 [-0.11, 0.18] | .07 | .65 |  | 0.01 [-0.11, 0.18] | .07 | .64 |
| *Education* |  |  |  |  |  |  |  |  |  |  |  |
| Less than college | -0.02 [-0.15, 0.07] | .06 | .52 |  | -0.01 [-0.13, 0.09] | .06 | .71 |  | -0.01 [-0.13, 0.09] | .06 | .72 |
| More than college | 0.04 [-0.03, 0.18] | .06 | .16 |  | 0.03 [-0.04, 0.17] | .05 | .26 |  | 0.03 [-0.04, 0.17] | .05 | .26 |
| *Employment* |  |  |  |  |  |  |  |  |  |  |  |
| Remote | 0.09 [0.08, 0.32] | .06 | .001 |  | 0.08 [0.06, 0.3] | .06 | .003 |  | 0.08 [0.06, 0.30] | .06 | .003 |
| Unemployed | 0.06 [0.02, 0.24] | .06 | .02 |  | 0.04 [-0.02, 0.2] | .06 | .12 |  | 0.04 [-0.02, 0.20] | .06 | .12 |
| Income | -0.04 [-0.09, 0.01] | .02 | .08 |  | -0.05 [-0.09, 0] | .02 | .05 |  | -0.05 [-0.09, 0.01] | .02 | .05 |
| Social desirability | 0.01 [-0.04, 0.05] | .02 | .85 |  | 0.02 [-0.02, 0.06] | .02 | .35 |  | 0.02 [-0.02, 0.06] | .02 | .35 |
| COVID-19 diagnosis | 0.01 [-0.33, 0.34] | .17 | .98 |  | 0.01 [-0.29, 0.36] | .17 | .82 |  | 0.01 [-0.29, 0.36] | .17 | .82 |
| Increased childcare | 0.02 [-0.10, 0.23] | .08 | .43 |  | 0.03 [-0.07, 0.25] | .08 | .27 |  | 0.03 [-0.07, 0.25] | .08 | .27 |
| Cohort | 0.03 [-0.02, 0.07] | .02 | .21 |  | 0.02 [-0.03, 0.06] | .02 | 0.44 |  | 0.02 [-0.03, 0.06] | .02 | .44 |
| Empathy | 0.23 [0.18, 0.27] | .02 | <.001 |  | 0.2 [0.15, 0.24] | .02 | <.001 |  | 0.20 [0.16, 0.25] | .02 | <.001 |
| Political ideology |  |  |  |  | 0.19 [0.14, 0.23] | .02 | <.001 |  | 0.19 [0.14, 0.23] | .02 | <.001 |
| Empathy X Political ideology |  |  |  |  |  |  |  |  | 0.01 [-0.03, 0.05] | .02 | .72 |
| R^2^ (R^2^ adjusted) | .07(.06) |  |  |  | .10(.09) |  |  |  | .10(.09) |  |  |
| Comparison | △R^2^ = .03, *F_(1,2018)_* = 71.45, *p* < .001 | | | | | |  |  |  |  |  |
|  |  |  |  |  | △R^2^ = .01, *F_(1,2017)_* = 0.13, *p* = .72 | | | | | |  |

*Note.* Age, social desirability, compassion, political ideology scores, and the belief that staying home prevents spread were standardized. *N* = 2036.

**S17 Output.** Predicting Whether One Stayed Home for Others among Liberal-leaning Participants with Logistic Regression (N=2695)


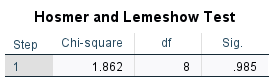


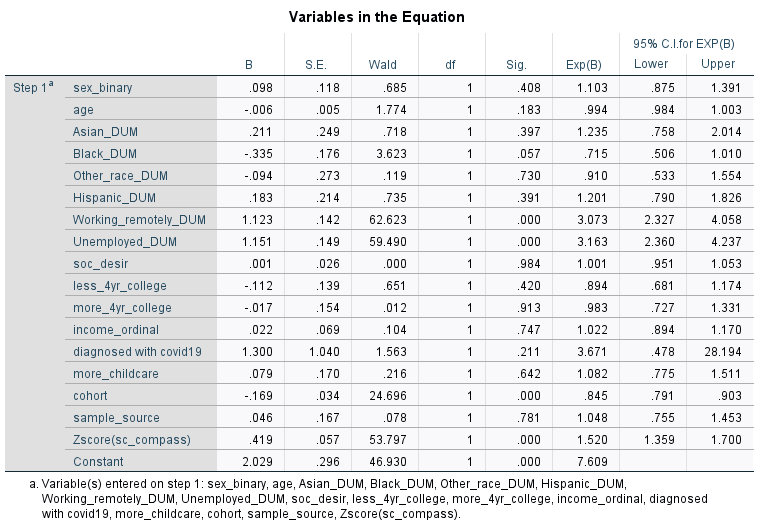


**S18 Output.** Predicting Whether One Stayed Home for Others among Independent Participants with Logistic Regression (N=872)


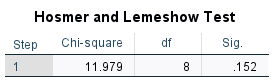


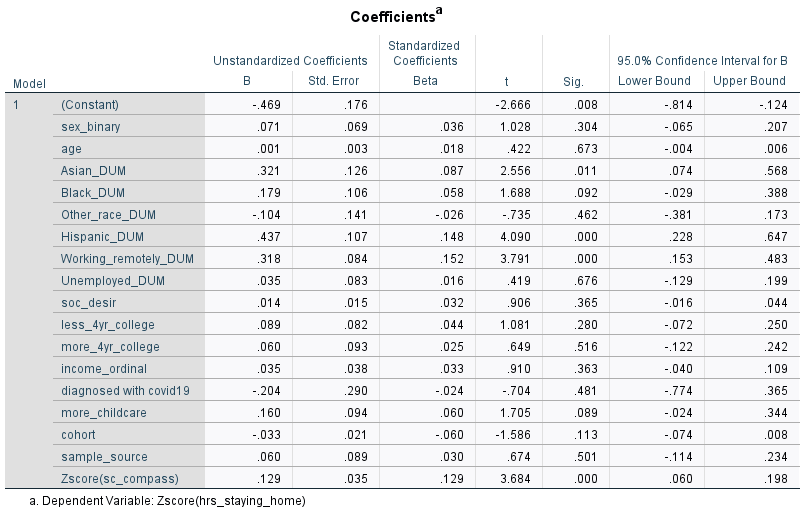


**S19 Output.** Predicting Whether One Stayed Home for Others among Conservative-leaning Participants with Logistic Regression (N=1966)


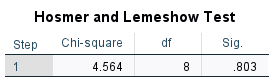


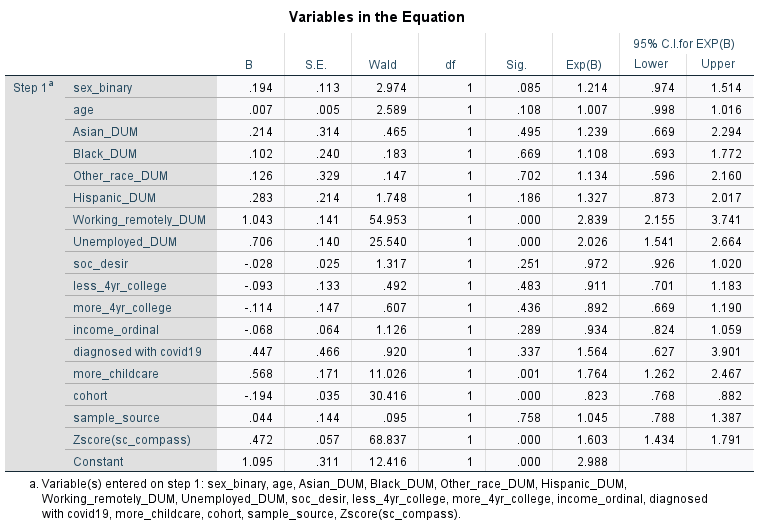


**S20 Output.** Predicting Time Spent Home among Liberal-leaning Participants with Multiple Regression (N=2695)


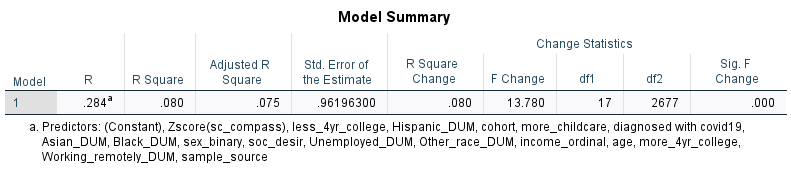


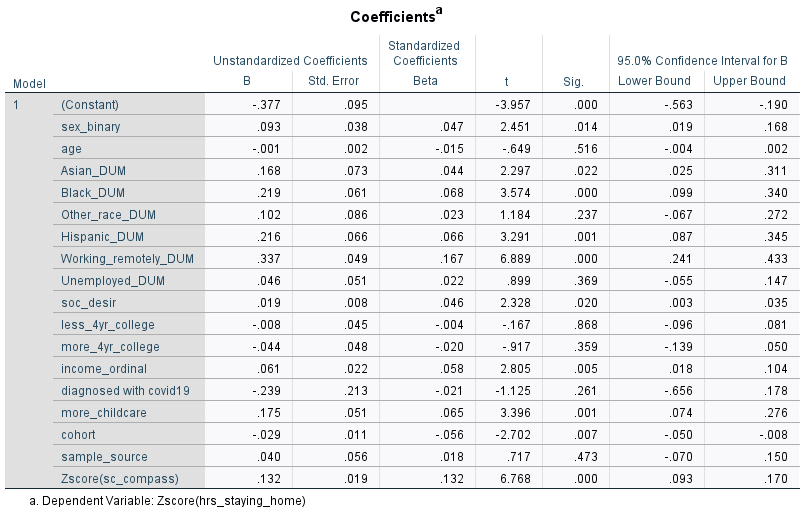


**S21 Output.** Predicting Time Spent Home among Independent Participants with Multiple Regression (N=872)


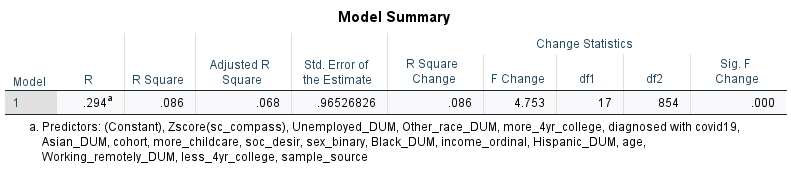


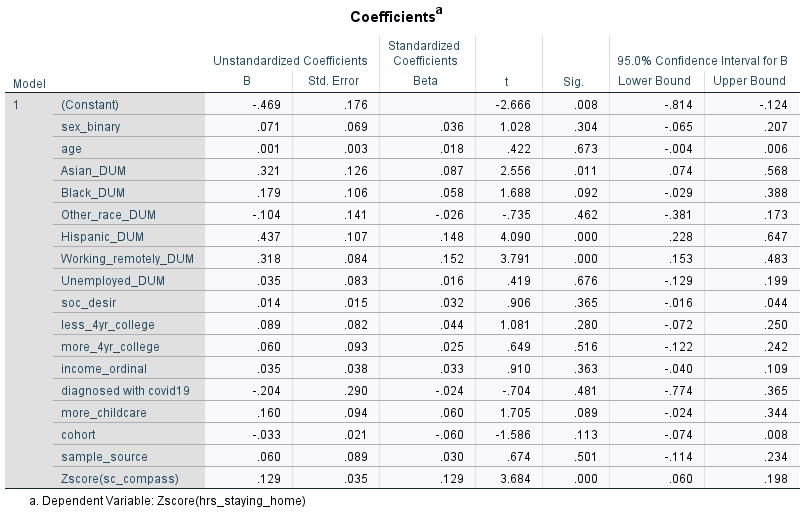


**S22 Output.** Predicting Time Spent Home among Conservative-leaning Participants with Multiple Regression (N=1966)


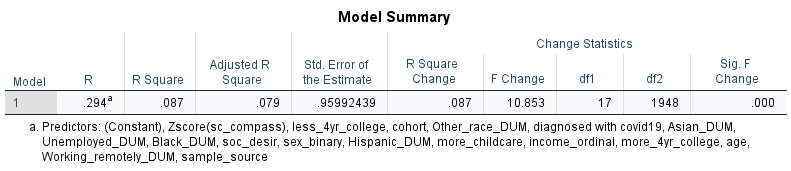

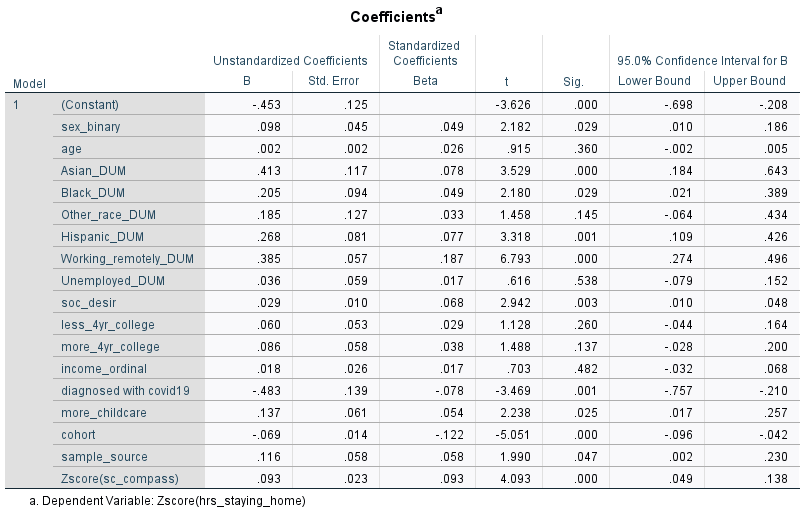


**S23 Output.** Predicting Time Spent Keeping a Distance among Liberal-leaning Participants with Multiple Regression (N=2695)
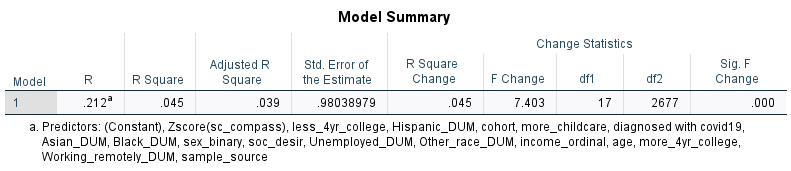

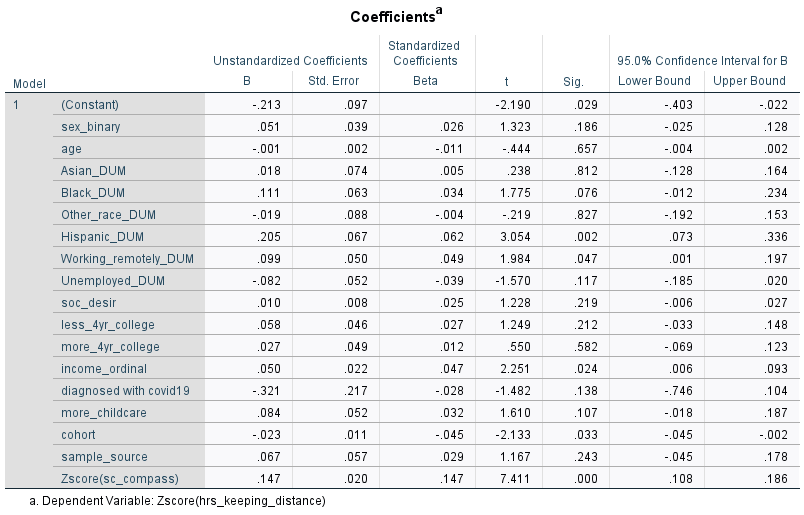


**S24 Output.** Predicting Time Spent Keeping a Distance among Independent Participants with Multiple Regression (N=872)


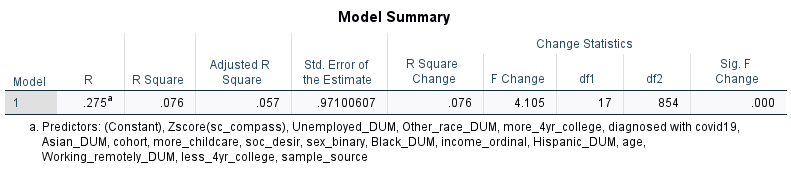


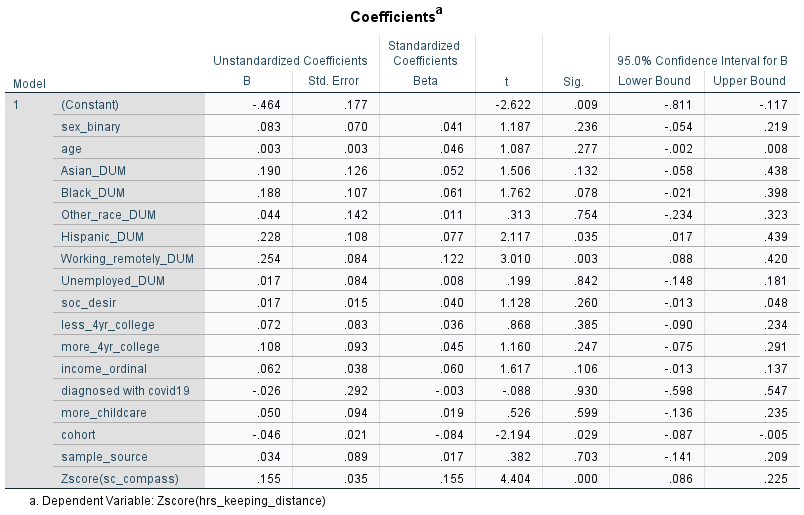


**S25 Output.** Predicting Time Spent Keeping a Distance among Conservative-leaning Participants with Multiple Regression (N=1966)


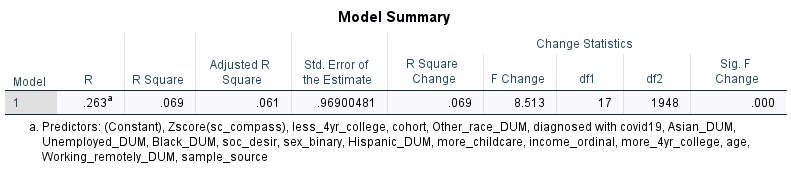


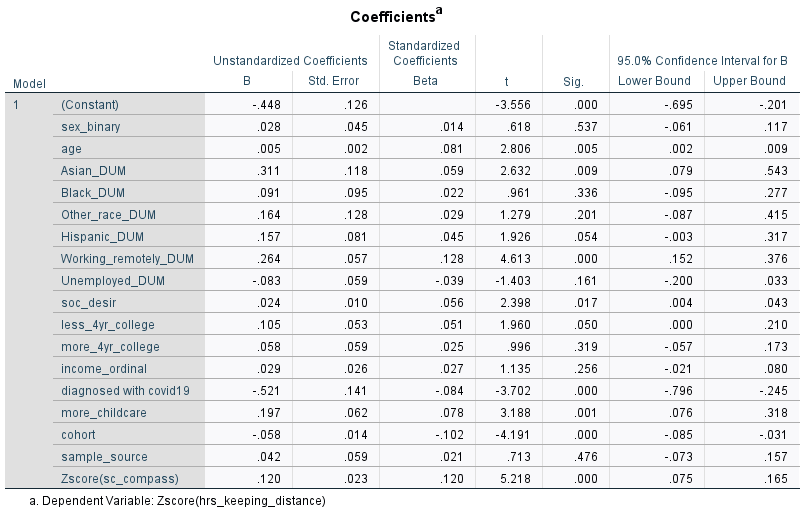


**S26 Output.** Predicting Mask Wearing among Liberal-leaning Participants with Multiple Regression (N=2695)


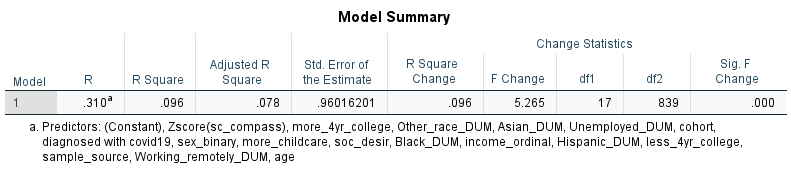


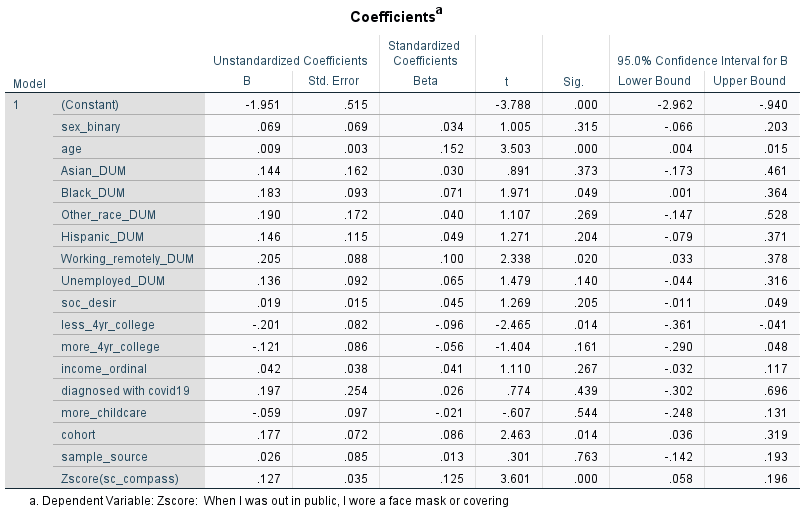


**S26 Output.** Predicting Mask Wearing among Independent Participants with Multiple Regression (N=343)


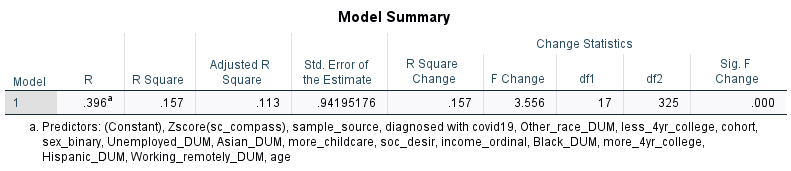


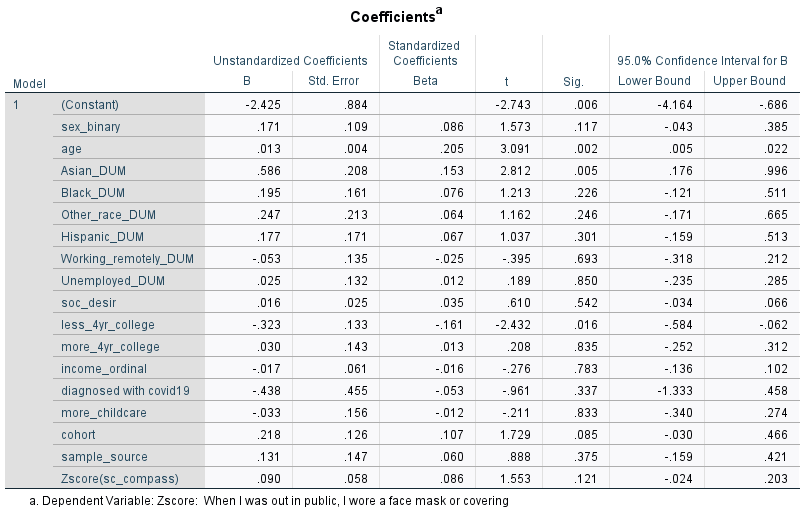


**S27 Output.** Predicting Mask Wearing among Conservative-leaning Participants with Multiple Regression (N=667)


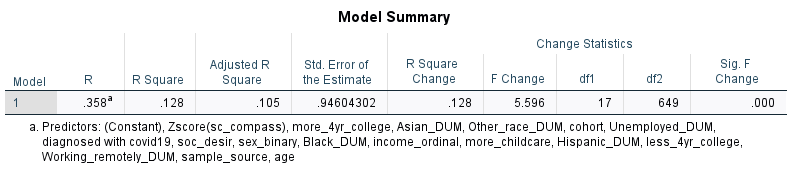


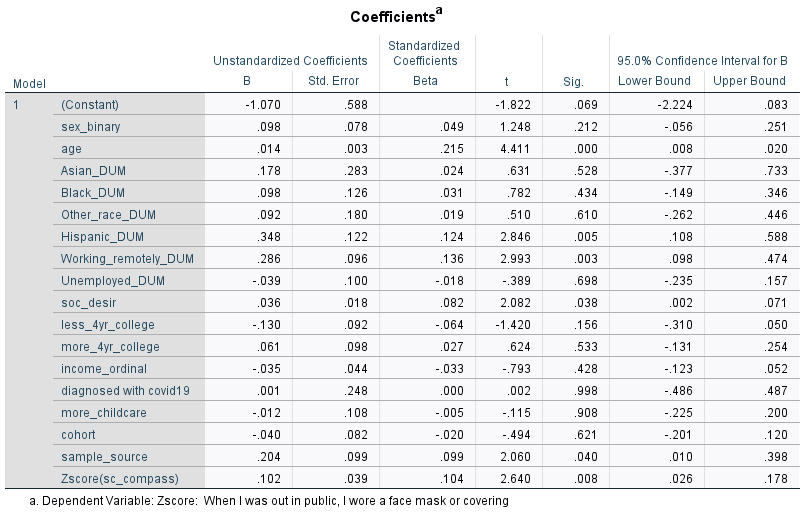


**S28 Output.** Predicting Understanding of Others’ Fear among Liberal-leaning Participants with Multiple Regression (N=2695)


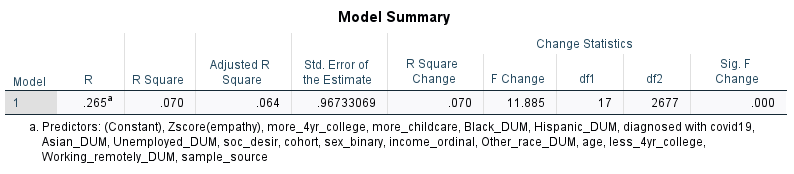


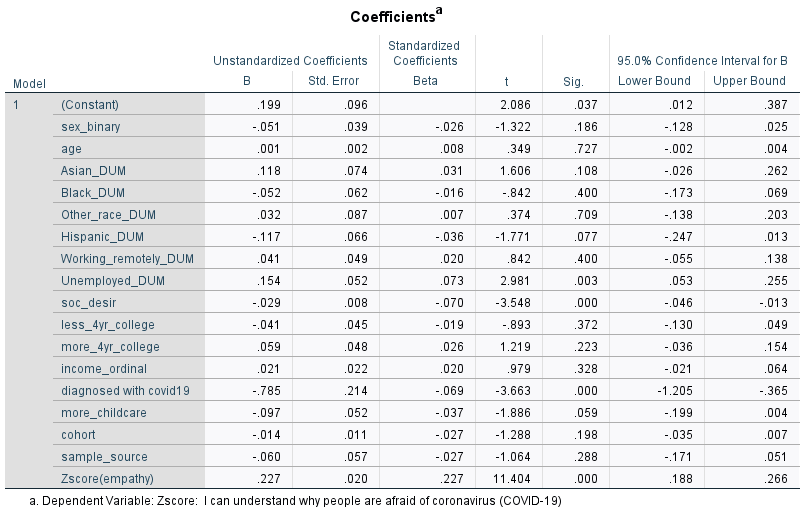


**S29 Output.** Predicting Understanding of Others’ Fear among Independent Participants with Multiple Regression (N=872)


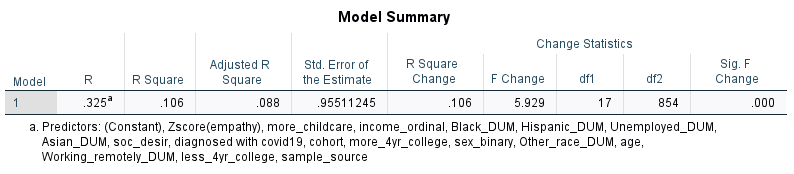


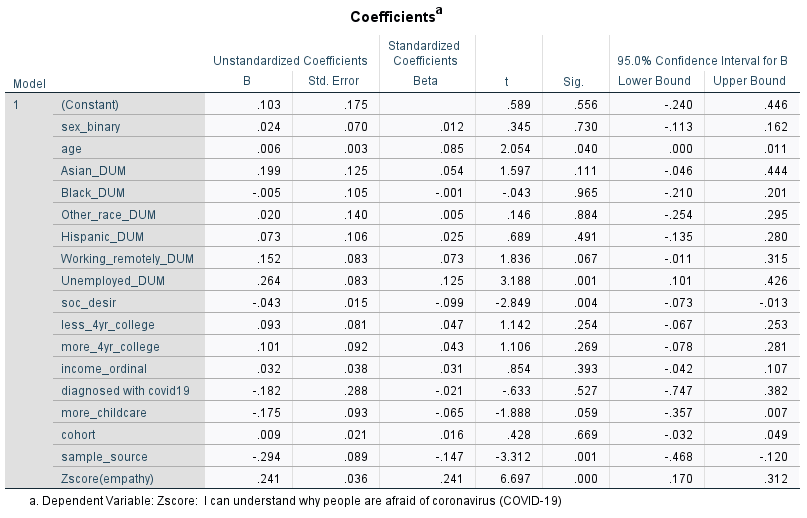


**S30 Output.** Predicting Understanding of Others’ Fear among Conservative-leaning Participants with Multiple Regression (N=1966)


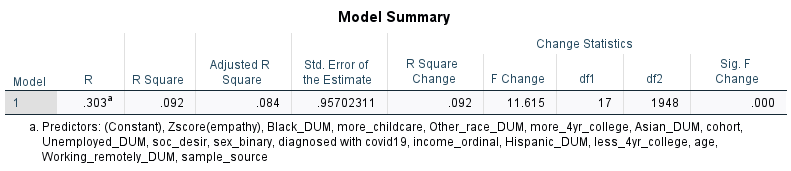


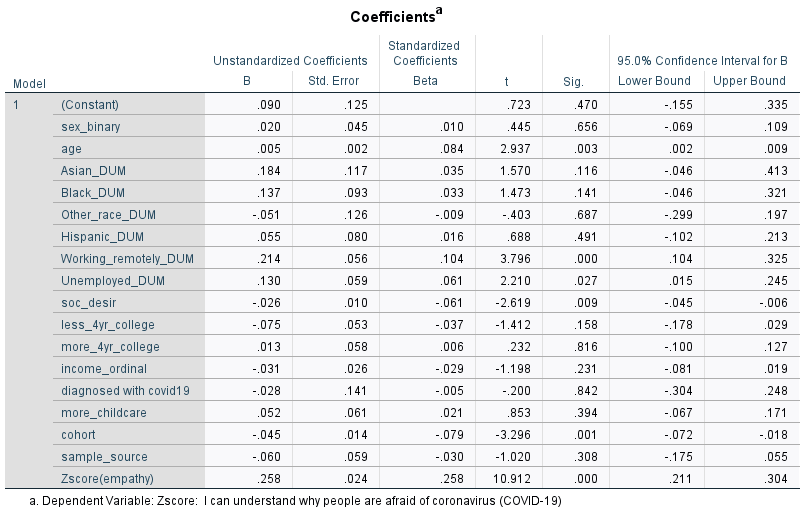


**S31 Output.** Predicting View of Others Overreacting among Liberal-leaning Participants with Multiple Regression (N=2695)


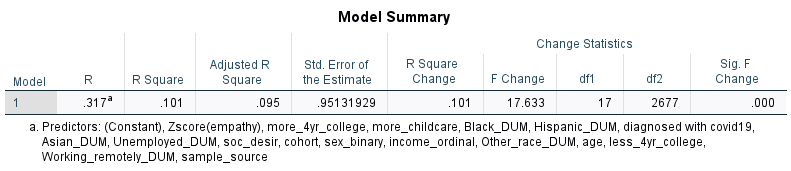


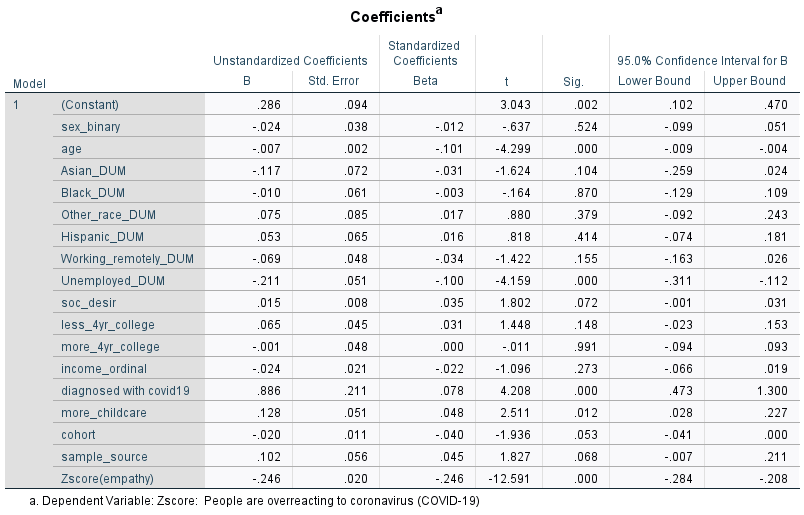


**S32 Output.** Predicting View of Others Overreacting among Independent Participants with Multiple Regression (N=872)


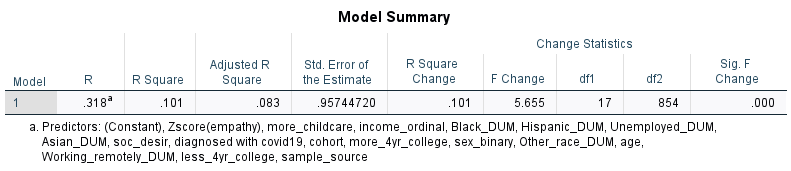


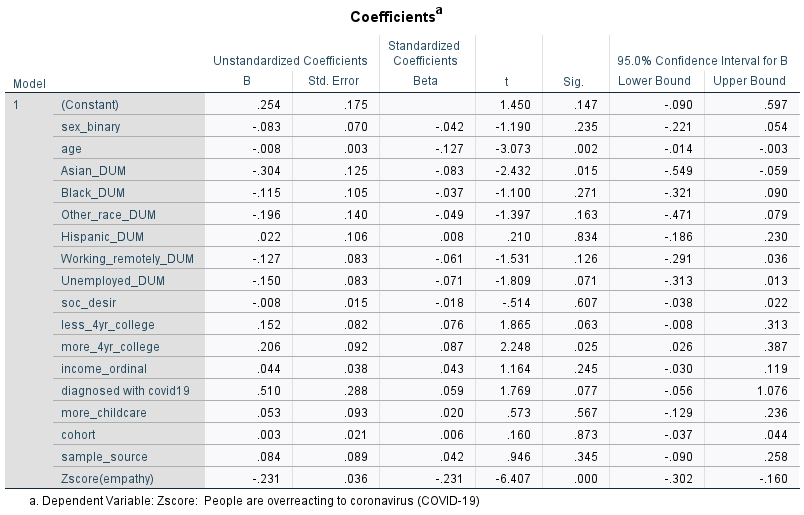


**S33 Output.** Predicting View of Others Overreacting among Conservative-leaning Participants with Multiple Regression (N=1966)


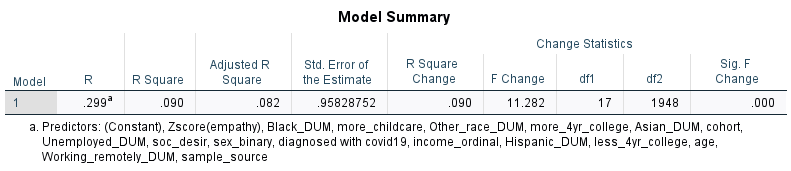


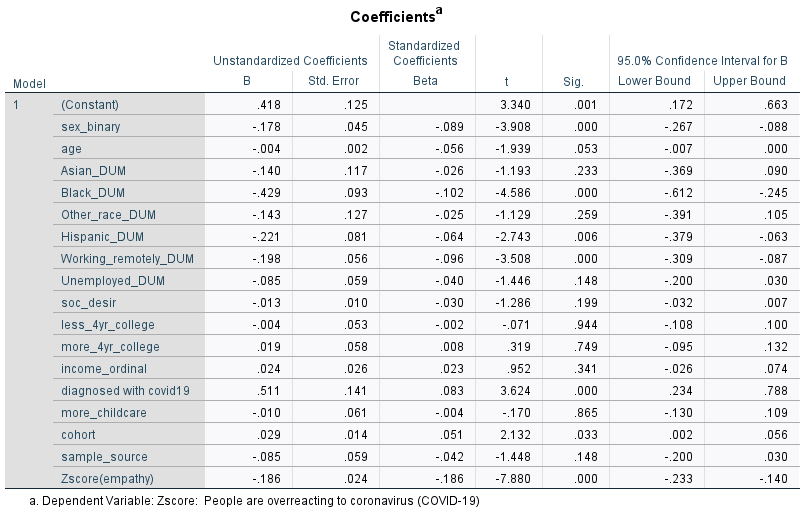


**S34 Output.** Predicting Belief that Staying Home Prevents the Spread among Liberal-leaning Participants with Multiple Regression (N=2177)


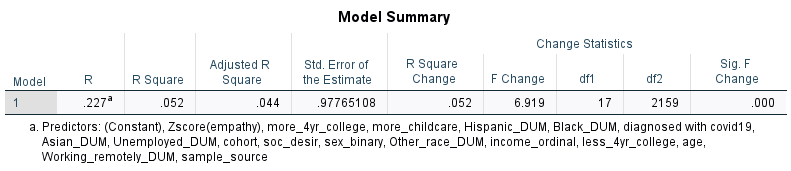


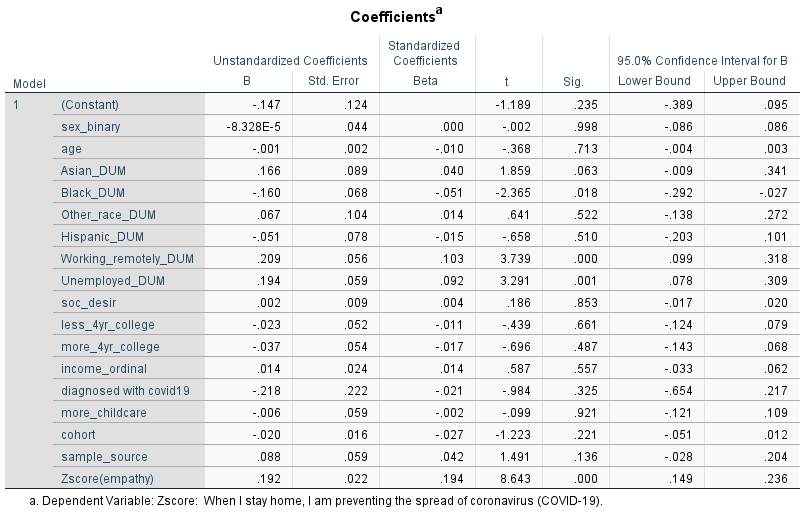


**S35 Output.** Predicting Belief that Staying Home Prevents the Spread among Independent Participants with Multiple Regression (N=753)


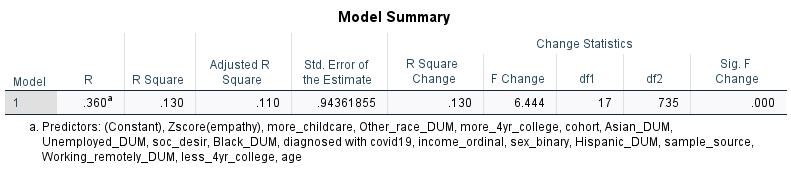


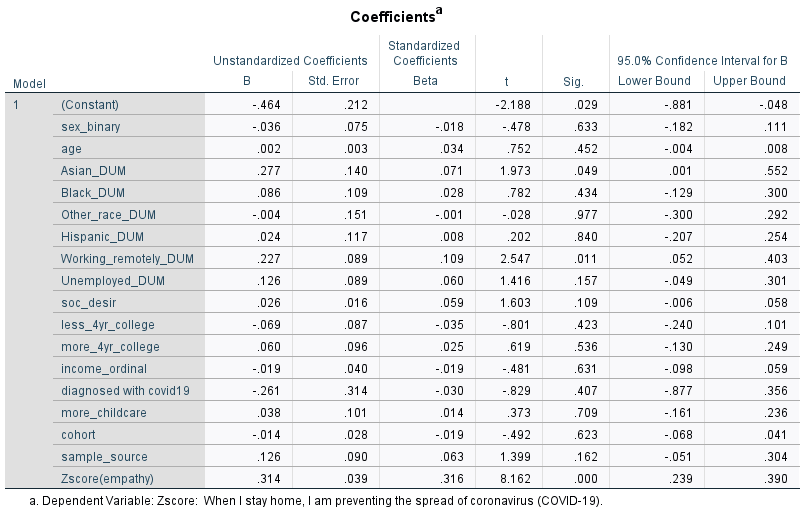


**S36 Output.** Predicting Belief that Staying Home Prevents the Spread among Conservative-leaning Participants with Multiple Regression (N=1705)


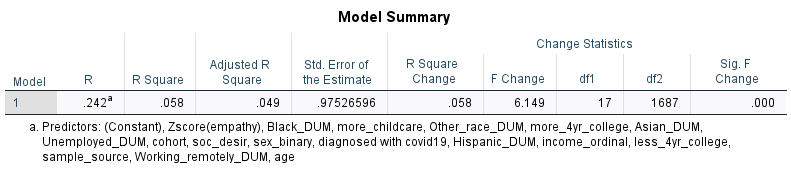


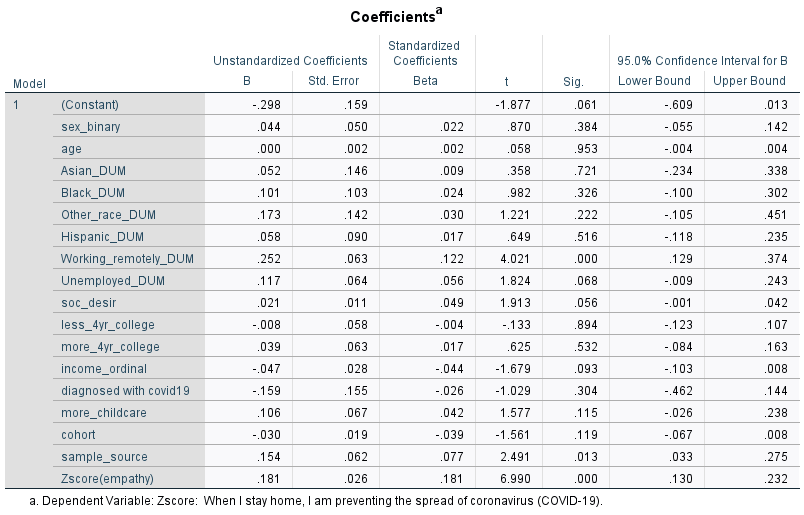


**S37 Table.** Correlations between Political Ideology and Prosocial Health Attitudes at Different Empathy Scores

|  | Empathy Score | | |
| --- | --- | --- | --- |
| Outcome Variable | 25^th^ Percentile (n=172) | Median  (n=236) | 75^th^ Percentile  (n=220) |
| Understanding of others’ fear | -.29*** | -.29*** | -.33*** |
| Viewing others as overreacting | -.42*** | -.41*** | -.47*** |
| Efficacy of sheltering in place | .26** | -.38*** | -.30*** |

*Note.* At the 25^th^ percentile of empathy, there were 69 conservative-leaning and 72 liberal-leaning participants. At the median of empathy, there were 81 conservative-leaning and 122 liberal-leaning participants. At the 75^th^ percentile of empathy, there were 76 conservative-leaning and 115 liberal-leaning participants. *** p < .01. *** p < .001.*

**S38 Table.** Correlations between Political Ideology and Prosocial Health Attitudes at Different Compassion Scores

|  | Compassion Score | | |
| --- | --- | --- | --- |
| Outcome Variable | 25^th^ Percentile (n=358) | Median  (n=391) | 75^th^ Percentile  (n=314) |
| Whether one stayed home for others | -.16*** | -.11*** | -.16*** |
| Time spent staying home | -.05*** | -.01*** | -.09*** |
| Time keeping distance from others | -.09*** | -.01*** | -.04*** |
| Frequency of mask wearing in public | -.29*** | -.22*** | -.37*** |

*Note.* For the three social distancing outcomes: at the 25^th^ percentile of compassion, there were 141 conservative-leaning and 134 liberal-leaning participants; at the median of compassion, there were 117 conservative-leaning and 209 liberal-leaning participants; and at the 75^th^ percentile of compassion, there were 110 conservative-leaning and 163 liberal-leaning participants. For the outcome of mask wearing: at the 25^th^ percentile of compassion, there were 43 conservative-leaning and 52 liberal-leaning participants; at the median of compassion, there were 43 conservative-leaning and 70 liberal-leaning participants; and at the 75^th^ percentile of compassion, there were 37 conservative-leaning and 48 liberal-leaning participants. *** p < .01. *** p < .001.*

**S39 Table.** Mean Scores and Standard Deviations for Empathy in the Total, MTurk and Qualtrics Samples, and for Empathic Concern in the Wave 22 of the 2008-2009 American National Election Studies (ANES) panel data

|  | Total Sample  N=5533 | MTurk Participants  N=3497 | Qualtrics Participants  N=2036 | ANES Sample  N=2237 |
| --- | --- | --- | --- | --- |
| Mean (*SD*) | 2.77 (*0.59*) | 2.81 (*0.61*) | 2.69 (*0.53*) | 2.89 (*0.66*) |

*Note*. Empathy was assessed by the Toronto Empathy Questionnaire using a 5-point Likert scale from never to always for actions indicating empathy (scale: 0-4). Empathic Concern was assessed by the Interpersonal Reactivity Index (Davis, 1983) using a 5-point Likert scale from strongly disagree to strongly agree (scale: 1-5). Even though one measure assessed frequency of empathy behaviors, while the other assessed agreement with statements describing the tendency to have empathic concern, both measures had 5-point Likert scales. Therefore, we present the mean for empathic concern scores from the ANES sample to match the scale of the Toronto Empathy Questionnaire, which was obtained by subtracting one from the mean (3.89) for empathic concern.
